# High Specific Strength Eutectic High‐Entropy Alloy: Collaborative Effects of TRIP, TWIP, and Nanoprecipitation

**DOI:** 10.1002/advs.202501703

**Published:** 2025-04-30

**Authors:** Z. Q. Wang, X. T. Li, Z. J. Zhang, Z. F. Zhang

**Affiliations:** ^1^ Shenyang National Laboratory for Materials Science Institute of Metal Research Chinese Academy of Sciences Shenyang 110016 P.R. China; ^2^ School of Materials Science and Engineering University of Science and Technology of China Shenyang 110016 China

**Keywords:** eutectic high‐entropy alloys, heterogeneous structure, nanoprecipitates, phase transformation, tensile strength, twinning

## Abstract

Eutectic high‐entropy alloys (EHEAs), characterized by their combination of hard and ductile phases, hold broad application prospects in terms of mechanical properties. However, the current performance of these alloys is not satisfactory. Herein, a new design approach is presented for EHEAs, focusing on precise composition regulation of each phase in the dual‐phase alloy. Hierarchically heterogeneous microstructure and integrating various strengthening mechanisms is successfully introduced such as phase transformation, twinning, and nanoprecipitates (NPs) into each single system. Finally, the overall strength and ductility are effectively enhanced. Specifically, the ultimate tensile strength is 1571 MPa, the uniform elongation is 22%, and the maximum strength can reach 2045MPa. Notably, the high Al content in the EHEA effectively reduces its density, resulting in the maximum specific ultimate tensile strength of 273 MPa cm^3^ g^−1^ in HEAs. The multi‐mechanism assisted strengthening (MMAS) strategy is expected to provide guidance for the design of dual‐phase alloys like EHEAs in the future.

## Graphical abstract

This study has designed a novel CoCrNiAl eutectic high‐entropy alloy with a two‐level heterogeneous structure with extremely high strength. The high mechanical properties originate from the collaborative strengthening mechanisms, including TWIP, TRIP, and nanoprecipitation. Due to the addition of a high Al content, the specific ultimate tensile strength reaches 273 MPa cm^3^ g^−1^, surpassing most existing structural metallic materials.

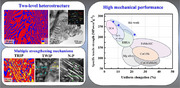

## 1 Introduction

As a vital pillar of modern industry, metallic materials are widely used in transportation, aerospace, navigation, nuclear power, architecture, electronics, and other fields.^[^ ^1^, ^2^, ^3^ ^]^ Enhancing the mechanical properties of metallic materials has become the key to breaking through industrial bottlenecks. Generally, effective methods to improve ductility include reducing stacking fault energy (SFE) for introducing work‐hardening mechanisms, as well as twinning‐induced plasticity (TWIP) or transformation‐induced plasticity (TRIP) effects.^[^ ^4^, ^5^ ^]^ However, these methods often struggle to achieve sufficient yield strength (YS).^[^ ^6^ ^]^ Alternatively, introducing high‐density defects like phase boundaries, grain boundaries, dislocations, or semi‐coherent or coherent nano‐precipitates (NPs) can effectively increase YS, but this typically comes at the expense of plasticity.^[^ ^7^, ^8^, ^9^, ^10^ ^]^ Therefore, finding a balance among various strengthening mechanisms is crucial for developing high‐performance metallic materials.

Eutectic high‐entropy alloys (EHEAs), emerging as a novel class of alloys in recent years, exhibit the characteristics of both eutectic alloys and high‐entropy alloys (HEAs) simultaneously. These EHEAs possess a unique dual‐phase structure, wherein the soft phase accommodates plastic deformation while the hard phase hinders dislocation movement, thereby achieving a favorable balance between strength and ductility. Furthermore, they retain the ease of casting associated with traditional eutectic alloys.^[^ ^11^, ^12^, ^13^, ^14^, ^15^, ^16^, ^17^ ^]^ Additionally, they typically have high‐Al content^[^ ^18^ ^]^ to reduce density, making them a potential new lightweight metallic material. Nevertheless, the current progress of EHEAs remains unsatisfactory, necessitating the development of superior alloys to meet growing industrial demands.

**Figure 1:**
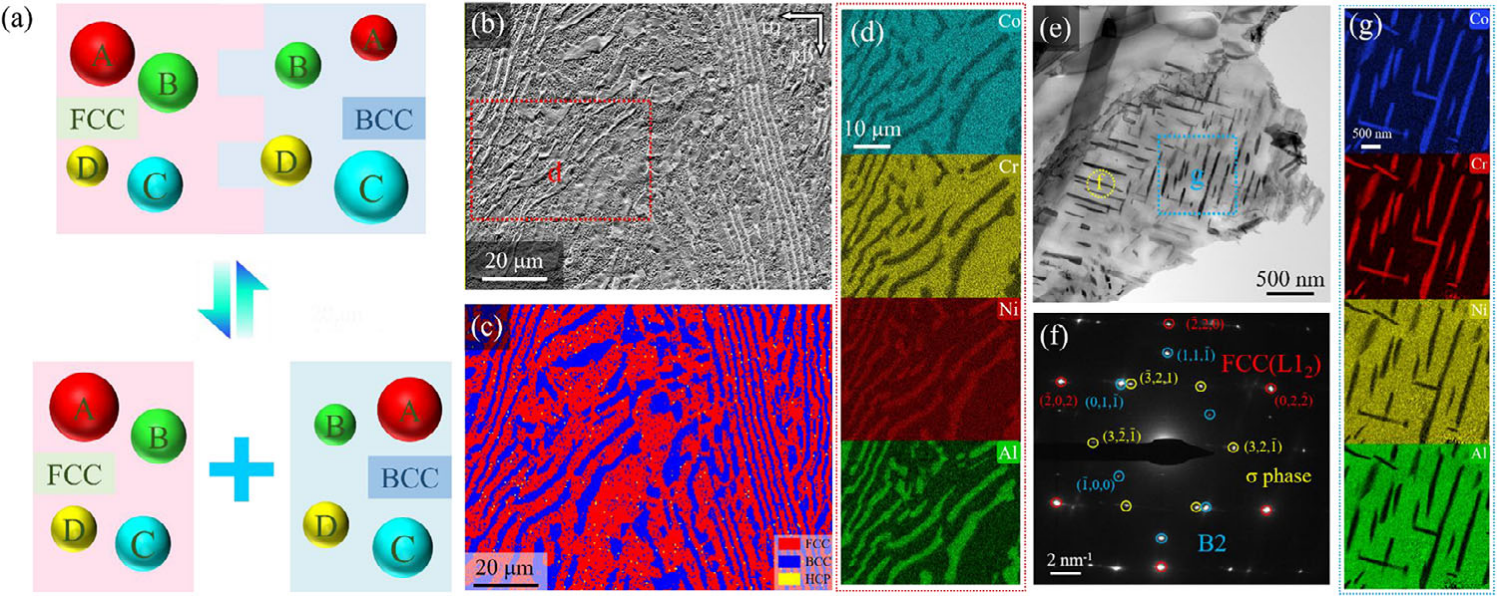
a) Schematic sketches of the design concept, mutual determination between biphasic components and total components. b) SEM image and c) EBSD phase maps showing the dual phase structure of EHEA. d) SEM‐EDS maps showing distribution of Co, Cr, Ni, and Al elements. e) Needle‐shaped nanoprecipitations (NPs) in BCC grains. f) Selected‐area electron diffraction (SAED) pattern of the circle in e) proved the existence of three different structures: L1_2_, B2, and σ phase. g) EDX maps of the blue frame in (e) illustrate the NPs rich in Co and Cr elements, while poor in Ni and Al elements.

Generally, the plastic deformation mechanisms of alloys are dominated by dislocation slip, twinning, as well as phase transformation.^[^ ^19^ ^]^ Here, we demonstrate how these mechanisms operate in an identical alloy with dual‐phase and how their synergistic effects enhance the mechanical properties of the alloy. As shown in **Figure** **1**a, typically, the design of dual‐phase alloys focuses on adjusting the overall composition. Conversely, can we achieve the desired overall composition by altering the composition in each phase with a special plastic deformation mechanism? Herein, we propose a novel design strategy for EHEAs, which involves precise composition regulation of each phase to elevate the overall performance. To simultaneously enhance the mechanical properties of both phases, controlling SFE is an effective approach. We selected a low‐SFE CoCrNi‐based EHEA with the addition of the Al element. By increasing Co and Cr content to decrease SFE and adjusting the dual‐phase composition, we obtained the desired overall composition through partitioning. Furthermore, the incorporation of Al led to the formation of a NiAl‐type B2 phase, which not only effectively increases strength but also reduces density, ultimately yielding a novel EHEA with the highest specific strength and good elongation in various HEAs. Specifically, we introduce the collaborative effects of TWIP and TRIP together by reducing the SFE of both phases, thereby enhancing great work‐hardening capability. We will display how the synergistic effect of multiple strengthening mechanisms can improve the mechanical properties of metallic materials, providing new ideas for the design of more duplex alloys in the future.

## 2 Results

### 2.1 Two‐Level Heterogeneous Structure

We develop an multi‐mechanism assisted strengthening (MMAS) EHEA with the chemical composition of Co_33_Cr_20_Ni_30_Al_17_ (at%) as described above. The as‐cast (AC) samples were annealed at 670, 730, and 810 °C after cold rolling (namely MMAS670, MMAS730, MMAS810, respectively, details see Materials and Methods), the heat treatment temperature is indicated by the differential scanning calorimetry (DSC) curve as shown in Figure S2 (Supporting Information). A unique two‐level heterogeneous microstructure was revealed as depicted in Figure 1. The micro‐scale structure is shown by the scanning electron microscope (SEM) image in Figure 1b, and the corresponding electron backscatter diffraction (EBSD) map in Figure 1c illustrates the typical dual‐phase structure with alternating face‐centered cubic (FCC) and body‐centered cubic (BCC) arrangements. The laminated BCC phase is embedded within the FCC matrix, accompanied by small, circular BCC phases formed during thermomechanical processing due to element redistribution.^[^ ^11^ ^]^ As the annealing temperature increased, the FCC matrix underwent a transformation from recovery to partial recrystallization, and finally to full recrystallization (Figure S3, Supporting Information). The energy dispersive spectroscopy (EDS) map, presented in Figure 1d and Figure S4a,b (Supporting Information), reveals that Ni and Al elements are generally enriched in the B2 phase, while Co and Cr elements are concentrated in the FCC matrix.

More detailed, we observed some nano‐scale heterogeneous structures where needle‐like NPs appeared within the BCC phase. The selected area electron diffraction (SAED) pattern in Figure 1f demonstrates the concurrent presence of three sets of diffraction patterns. The FCC structure of the matrix was identified as the Ni3Al‐type L1_2_ phase, while the BCC structure was determined to be the NiAl‐type B2 phase. Uniquely, the NPs were indexed as σ phase with the tetragonal crystal structure. The EDS map of the blue frame in Figure 1e has been shown in Figure 1g and Figure S4c,d (Supporting Information), where the NPs also exhibit enrichment of Co and Cr elements. The formation of the σ phase in HEAs is believed to be closely related to the valence electron concentration (VEC).^[^ ^20^ ^]^ When the VEC ranges from 6.88 to 7.84, the σ phase may appear in the AC or annealed samples. Additionally, the supersaturation of Co and Cr elements in the B2 phase promotes the precipitation of the Co‐ and Cr‐rich σ phase.^[^ ^19^, ^21^ ^]^ High‐resolution transmission electron microscopy (HR‐TEM) images in Figure S5 (Supporting Information) reveal lattice mismatches between the NPs and the B2 phase, along with geometrically necessary dislocations (GNDs) at the interface to maintain lattice continuity. Through statistical analysis of the TEM images (more than 10 images) in Figure S6 (Supporting Information), it was observed that the length and width of the NPs gradually increased with the annealing temperature, but there were no significant changes in their morphologies.

As illustrated in Figure S7a–c (Supporting Information), following rolling and annealing treatments, the B2 phase predominantly adopts (111) orientations, while the (101) crystal planes are more frequently observed in the L12 phase. In contrast, the AC samples exhibited coincident points between the (111)_L12_ and (110)_B2_ orientations, as well as between the (110)_L12_ and (111)_B2_ orientations (Figure S7d–f, Supporting Information), indicating a typical Kurdjumov‐Sachs (K‐S) orientation relationship,^[^ ^22^ ^]^ in which {111}_FCC_||{110}_BCC_, <110>_FCC_||<111>_BCC_ between the two phases. Nevertheless, it is crucial to emphasize that the orientation distribution is more concentrated in the annealed state compared to the AC samples, which is consistent with other results.^[^ ^14^ ^]^ The influence of this preferred orientation on the mechanical properties will be analyzed in the later section.

### 2.2 Mechanical Properties

**Figure 2:**
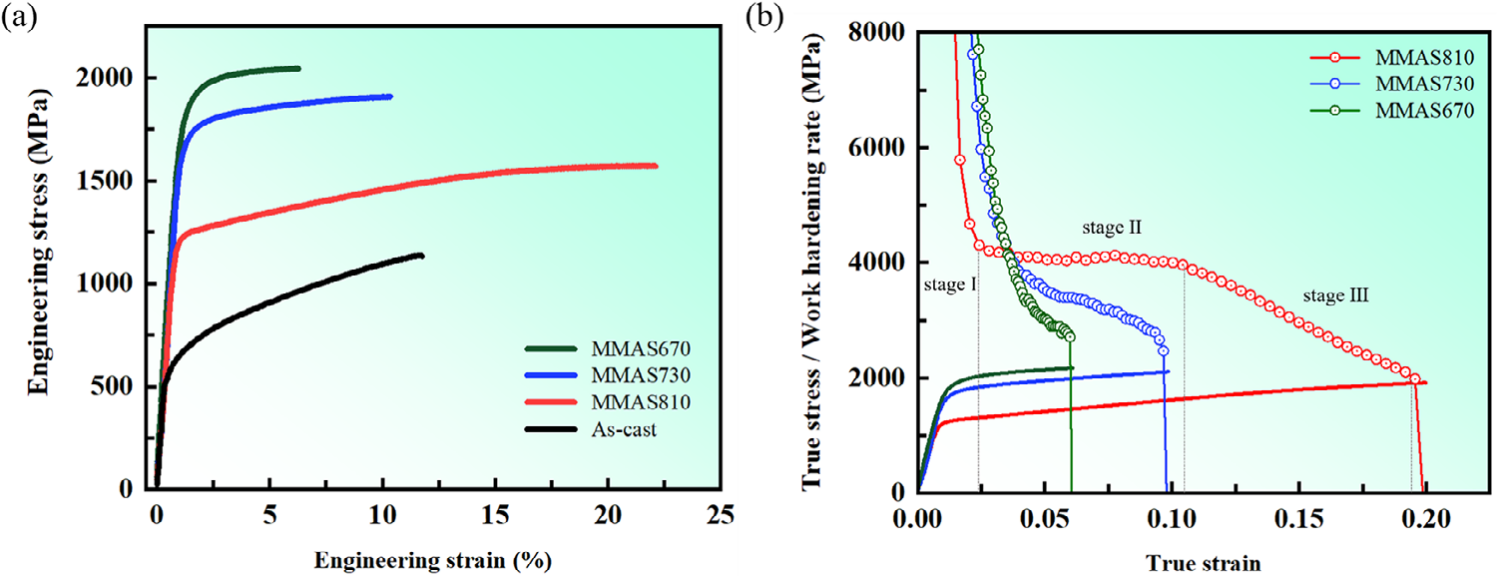
Mechanical properties of EHEA prepared by different thermomechanical processing at room temperature. a) Tensile engineering stress‐strain curves of the MMAS670, MMAS730, MMAS810, and AC samples. b) Strain‐hardening rate versus true strain demonstrated the increase in work‐hardening rate with the increase of annealing temperature, ultimately showing three‐stage behavior in MMAS810 sample.

Our design strategy of combining a two‐level heterogeneous structure with low SFE, has led to significant improvements in mechanical properties. As demonstrated by the uniaxial tensile stress‐strain curves at room temperature presented in **Figure** **2**a, the MMAS810 sample exhibits an excellent combination of YS (1218 MPa), ultimate tensile strength (UTS) (1571 MPa), and uniform elongation (UE) (22%). As for the MMAS670 and MMAS730 samples, due to the effect of high‐density dislocations, there is still a trade‐off relationship between strength and ductility, with a maximum strength 2045 MPa while maintaining a certain level of ductility. In contrast, the AC sample displays a shortage in neither strength nor ductility. These results demonstrate the crucial role of microstructural refinement and orientation selection during thermo‐mechanical processing in enhancing the mechanical properties.

The true stress‐strain and work‐hardening curves are presented in Figure 2b. The work‐hardening rate (WHR) exhibits a classical three‐stage process. In the stage I, the WHR rapidly decreases due to the annihilation of screw dislocations.^[^ ^23^ ^]^ Then the WHR remains nearly constant in stage II, indicating the occurrence of certain work‐hardening mechanisms. Finally, in stage III, the WHR begins to gradually decrease due to the massive annihilation of dislocations and the depletion of work‐hardening capacity. In the fully recrystallized MMAS810 sample, the intersection of the WHR and true stress‐strain curves satisfies the classical Considère criterion, where the intersection point represents the necking point.^[^ ^24^ ^]^ However, a catastrophic fracture occurs immediately after necking, which is indicated by the peak of the engineering stress‐strain curve, suggesting inadequate intrinsic ductility. For the other MMAS730 and MMAS670 samples, the WHR remains above the true stress‐strain curve until the final moment, when a sudden drop in the WHR intersects with the true stress‐strain curve, and then leading to the occurrence of necking. This indicates a micro‐scale cracking dominated fracture mechanism.^[^ ^25^ ^]^

### 2.3 Deformation Mechanisms

**Figure 3:**
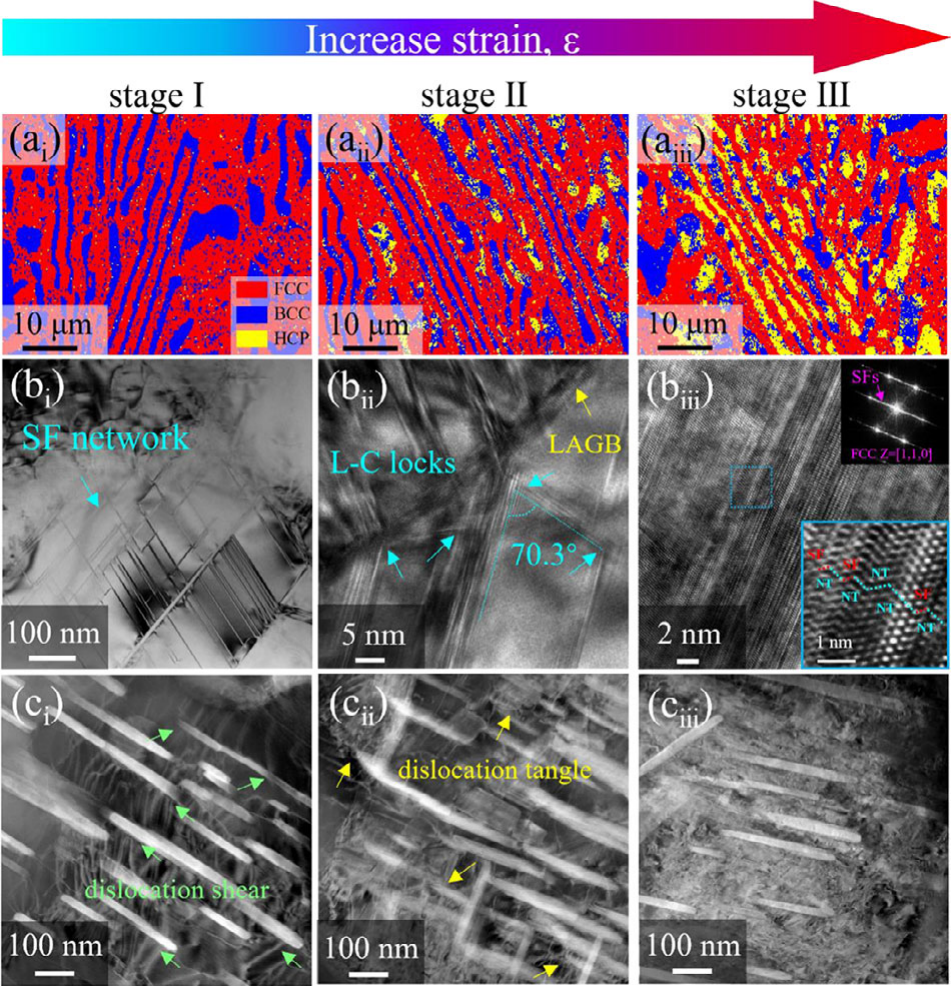
The deformation mechanism of MMAS EHEA indicated the collaborative effects of TWIP, TRIP, and NP in three stages. (a_i_‐a_iii_) The EBSD phase maps show the phase transition from BCC to HCP during loading. (b_i_‐b_iii_) The evolution of twins and SFs at three stages. (b_i_) High‐density SF networks in FCC matrix. (b_ii_) L‐C locks formed on both sides of the low angle grain boundary (LAGB), and the included angle between the two atomic planes is determined to be 70.3°. (b_iii_) HR‐TEM image showing the final stage (9%) of atomic surface arrangement, the inset displays the corresponding fast Fourier transform (FFT) pattern, and the larger image of the blue frame explains the mutual stacking of SFs and nanotwins (NTs). (c_i_‐c_iii_) High‐angle annular dark field (HAADF)‐TEM image showing the interaction between dislocations and NPs. (c_i_) The shearable nature of dislocations at the early stage. (c_ii_) The formation of dislocation tangles after dislocation multiplication. (c_iii_) High‐density dislocations piled‐up in the grain.

To better understand the deformation mechanisms during the three stages, EBSD and TEM were conducted on the MMAS810 sample. We could observe the transformation of B2 lamellae from BCC to HCP structure, as shown in **Figure** **3**a_i_‐a_iii_, indicating a typical TRIP effect. In the final stage of loading, the volume fraction of phase transformation in the B2 phase reached up to 48.0%. The corresponding Kernel average misorientation (KAM) map is shown in Figure S8 (Supporting Information). In Stage I, the internal stress is mainly concentrated in the B2 phase, while the FCC matrix exhibits a fully recrystallized state with low stress. Subsequently, the internal stress in the FCC phase rapidly increases but remains lower than that in the B2 phase until the occurrence of fracture. On the other hand, as shown in Figure 3b_i_‐b_iii_, TWIP effect was observed in the FCC matrix. In Stage I as shown in Figure 3b_i_, the overall plastic deformation has just begun, but in the soft FCC phase, local stress has reached a high level, so high‐density stacking fault (SF) networks are activated, resulting in a pronounced dynamic structural refinement effect.^[^ ^26^ ^]^ In Figure 3b_ii_, high‐density SFs can be observed on both sides of the low‐angle grain boundary (LAGB), as well as immobile Lomer‐Cottrell (L‐C) locks formed when two SFs meet each other. The angle between the two atomic planes is determined to be 70.3°. Furthermore, prior to fracture, the misalignment of atomic planes underwent sufficient development, forming a structure where nano‐twins and SFs are stacked together (Figure 3b_iii_). In particular, the FFT pattern in the upper right corner exhibits strong SF characteristics, which obscure the spots of the twins. Furthermore, the interaction between NPs and dislocations is illustrated in Figure 3c_i_‐c_iii_. Multiple dislocations cutting through needle‐shaped NPs can be observed in Figure 3c_i_. Although the size of the NPs significantly increases during annealing, the dislocation cutting mechanism is still maintained. Compared to the bypassing mechanism, the cutting mechanism is less likely to cause stress concentration due to pilling‐up of dislocations.^[^ ^27^ ^]^ This typically occurs when the precipitate size is small and the interfacial mismatch is low.^[^ ^28^ ^]^ As dislocations continue to multiply, they interact with each other, forming more dislocation tangles. Finally, a high density of dislocations accumulated within the matrix.

To further support the previous findings, the electron channel contrast (ECC) images in Figure S9 (Supporting Information) enable simultaneous observation of the synergistic effects of multiple mechanisms. We can observe the transformation from a few twins to parallelly arranged twins, and then to multi‐level twins in the FCC matrix. Additionally, NPs and lamellar HCP phases also contribute to the improvement of mechanical properties in the B2 phase. Regarding the MMAS670 (Figure S10, Supporting Information) and MMAS730 (Figure S11, Supporting Information) samples, despite the presence of high‐density dislocations, microstructures such as twins, SF networks, and L‐C locks can still emerge when the stress exceeds the critical value, enhancing the work‐hardening capability. It is worth noting that in this dual‐phase structural material, combining both soft and hard phases, when subjected to external loads, as illustrated by Figure S12 (Supporting Information), the soft domain first exceeds the yield point and undergoes plastic deformation. The resulting GNDs pile‐up at the phase boundaries and exert a force on them. Conversely, the hard domain also exerts a reaction force on the soft domain. This interaction provides additional hardening, known as hetero‐deformation induced (HDI) hardening.^[^ ^29^ ^]^ It is important to note that in traditional homogeneous structures, the back stress is not remarkable enough so that HDI hardening is not the primary strengthening mechanism. However, in the heterogeneous structured metallic materials, HDI hardening can provide an additional strengthening effect due to the dramatic variation in flow stress between the two domains, which makes it not be ignored.

## 3 Discussion

**Figure 4:**
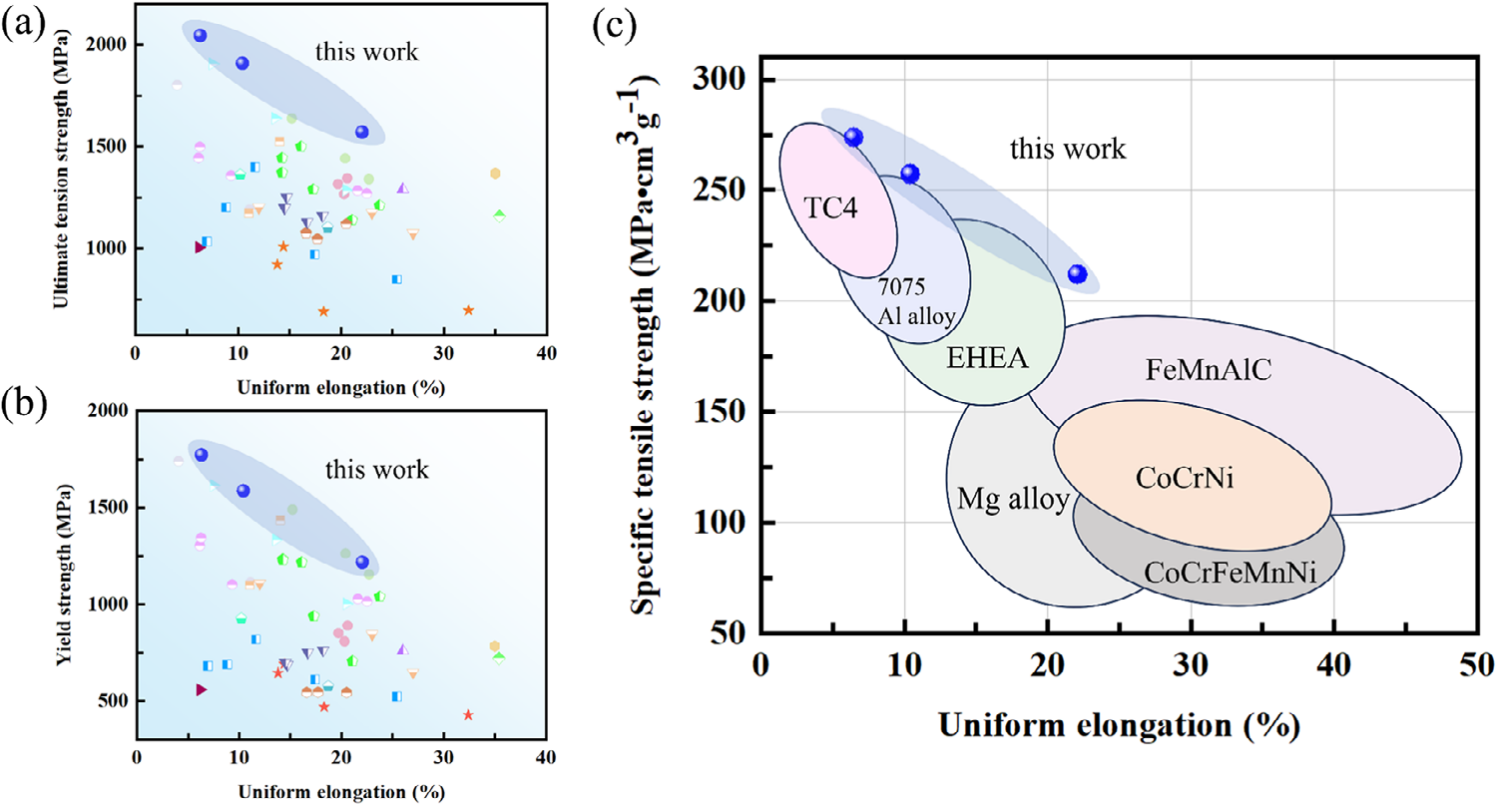
Comparison of a) YS and b) UTS versus UE with other EHEA in literature. c) Comparison of specific ultimate tensile strength (SUTS) versus UE with lightweight engineering alloys. These results indicate that our EHEA successfully breaks the trade‐off relation of strength and ductility of the existing EHEAs.

We compared the mechanical properties of MMAS EHEA with other alloys reported in the literature. **Figure** **4**a,b illustrates the comparison of YS and UTS versus UE between our MMAS EHEA and other EHEAs available (Detailed data can be found in Table S1, Supporting Information), respectively. Our MMAS strategy exhibits a superior combination of both strength and ductility, surpassing most EHEAs reported in the literature. In particular, due to the addition of a high content of Al element, MMAS EHEAs typically have a lower density (7.49 g cm^−3^) compared to traditional materials, resulting in ultra‐high specific ultimate tensile strength (SUTS) (up to 273 MPa cm^3^ g^−1^) for our MMAS EHEA. Therefore, as shown in Figure 4c (Detailed data can be found in Table S2, Supporting Information), our MMAS EHEAs demonstrate a better relationship between SUTS and UE compared to most lightweight engineering structural materials in the world. These results demonstrate the significant role of collaborative strengthening strategy in enhancing the performance of heterogeneous structural materials.

**Figure 5:**
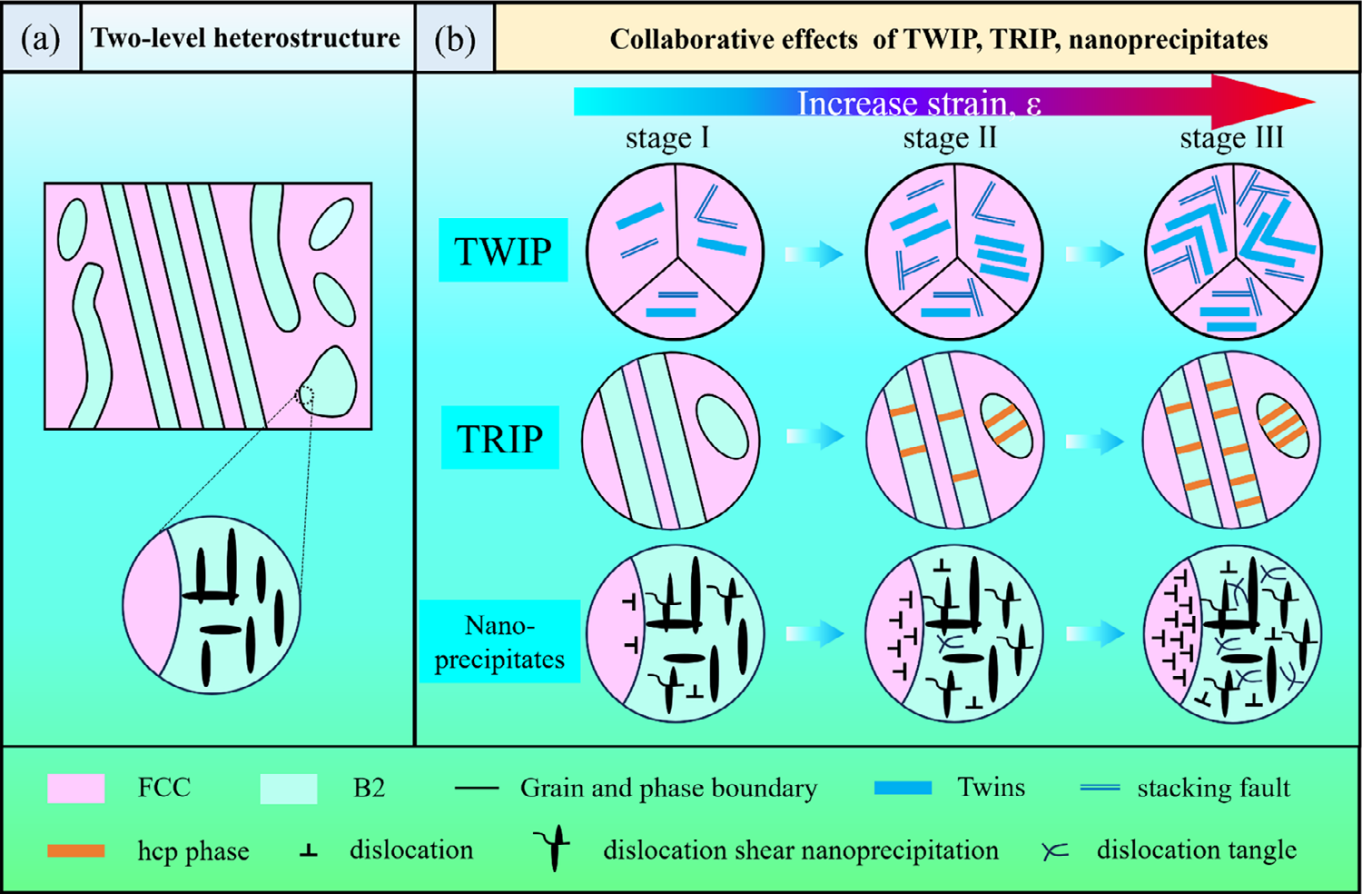
Schematic diagram of the origin of the heterogeneous structure and multi‐mechanism in the MMAS EHEA. a) Schematic of a two‐level heterostructure. b) Summary of deformation mechanisms: TWIP, TRIP, and nanoprecipitates.

Based on the experimental results obtained, the evolution of microstructure and deformation mechanisms are schematically represented in **Figure** **5**. Multiple mechanisms play different roles in improving its mechanical properties. First, the high YS exceeding 1.2 GPa originates from the unique two‐level heterogeneous structure as displayed by Figure 5a. During the loading process, the soft FCC phase reaches the yield point first, while the hard B2 phase remains in an elastic state. To maintain strain continuity, a stress gradient, known as the long‐range back stress, emerges near the phase boundaries due to the accumulation of GNDs.^[^ ^29^ ^]^ This HDI strengthening elevates the stress state within the soft phase and makes it achieve a higher stress level, thereby enhancing the overall YS.^[^ ^11^, ^30^, ^31^ ^]^ Additionally, precipitation strengthening contributes significantly to the YS. The interaction between coherent or semi‐coherent NPs with dislocations also brings about an orderly strengthening effect ^[^ ^8^, ^32^ ^]^ effectively increasing the yield strength of the B2 phase and, consequently, the overall YS.

Second, as shown in Figure 5b, the high ductility of the MMAS EHEA originates from the combined contributions of phase transformation, twinning, as well as the interaction between NPs and dislocations. In the initial stage of strain, less than 4%, plastic deformation is primarily dominated by twinning and dislocations. Subsequently, phase transformation gradually occurs and becomes one of the dominant strengthening mechanisms. Both TWIP and TRIP effects can be triggered by low SFE, which tends to make dislocations move on different slip planes separated by twins and phase boundaries, effectively avoiding stress concentration. When the critical stress is exceeded, nano‐twins and HCP lamellae are formed. When two Shockley partial dislocations on different (111) atomic planes encounter each other, an immobile L‐C locks can be formed.^[^ ^33^ ^]^ All these microstructures constitute a dynamic microstructure refinement effect. On the one hand, they hinder dislocation movement, and on the other hand, dislocations can only move along the channels of the microstructures, dissipating stress concentration. Furthermore, it has been reported that these microstructures can also serve as sources of dislocation multiplication and thus enhance the work‐hardening capability.^[^ ^33^ ^]^ However, excessively low SFE may not be ideal, resulting in unsatisfactory mechanical properties of the alloy at liquid nitrogen temperature (Figure S13, Supporting Information). Although there is no significant difference in deformation mechanisms compared to room temperature (Figure S14, Supporting Information), we speculate that excessively rapid plastic deformation caused by excessively low SFE may lead to premature catastrophic fracture. Additionally, the interaction between NPs and dislocations is also believed to contribute to the high ductility at a very high strength level. NPs can act as obstacles to dislocation movement, and the cutting mechanism is less likely to cause stress concentration, thereby not excessively damaging plasticity.^[^ ^34^ ^]^ Moreover, it has been reported that NPs can also serve as sources of dislocation multiplication, further enhancing the work‐hardening capability.^[^ ^28^, ^35^ ^]^ It can be concluded that our MMAS strategy can effectively improve the comprehensive mechanical properties of this type of dual‐phase alloy by the collaborative effects of TWIP, TRIP, and nanoprecipitation.

Finally, we observed the damage morphology on the side of the MMAS810 sample in Figure S15 (Supporting Information). In the final stage of plastic deformation, numerous cracks appeared near the B2 phase. Among them, most of the cracks penetrated the B2 phase (red arrow), while the rest cracks were along the phase boundary (green arrow) or inserted into the B2 phase (yellow arrow). This indicates the hard heterogeneous phase has a dual impact. On the one hand, it effectively increases the overall YS by inducing the HDI hardening in the initial stage of plastic deformation.^[^ ^29^ ^]^ On the other hand, in the final stage of plastic deformation, the hard phase undergoes sequential cracking, becoming a source of crack propagation. However, it is worth mentioning that due to the presence of the ductile FCC phase, cracks cannot rapidly propagate and cause catastrophic failure. It should be emphasized that the heterogeneous structure combining hardness and ductility effectively integrates the advantages of both phases. Moreover, we discovered some interesting phenomena in the AC samples (Figure S16, Supporting Information). The (001), (101), and (111) orientations within the B2 phase exhibited distinct phase transformation tendencies, as illustrated in Figure S16e (Supporting Information). The reduction ratios for these orientations are 96.2%, 79.1%, and 44.1%, respectively. The (001) orientation had almost phase transition completely and (111) orientation showed the lowest degree of phase transformation, highlighting the orientation dependence of the TRIP effect. By considering the evolution of texture during thermo‐mechanical processing (Figure S7, Supporting Information), we identified another crucial factor contributing to improved performance. The rolling and annealing processes eliminated most of the easy‐to‐transform orientation, allowing phase transformations to occur slowly and sustainably. This provided continuous work‐hardening capability, thereby delaying the onset of fracture. It should be noted that the selection of appropriate thermo‐mechanical processes can not only refine the microstructure and eliminate defects, but also, is often overlooked, filter out undesirable orientations.

## 4 Conclusions 

In this study, we propose a novel MMAS strategy for the design of EHEAs through the collaborative effects of TWIP, TRIP, and nanoprecipitation. By introducing multiple strengthening mechanisms such as phase transformation, twinning, and NPs into an identical alloy system, we effectively enhance both the strength and ductility, achieving UTS of 1571 MPa, UE of 22%, and maximum strength 2045 MPa. The HDI hardening and evolution of texture during thermomechanical processing also play a significant role in enhancing the mechanical performance. Additionally, our MMAS EHEA exhibits an ultra‐high SUTS due to the high‐Al content, with the maximum value of 273 MPa cm^3^ g^−1^. The dual‐phase alloy strengthened by NiAl phase shows promise as a highly prospective lightweight alloy. We believe that the precise composition regulation of each phase in EHEAs will facilitate the design of alloys with superior mechanical performance in the future, and this strategy has the potential to be extended to other alloy systems.

## 5 Materials and Methods

Materials and methods can be found in the supporting information.

## Conflict of Interest

The authors declare no conflict of interest.

## Author Contributions

Z.Q.W. did conceptualization, data curation, investigation, methodology, visualization, writing original draft, writing review & editing. X.T.L. did the methodology, investigation, and visualization. Z.J.Z did the methodology, investigation, and supervision. Z.F.Z did conceptualization, funding acquisition, project administration, supervision, resources, validation, visualization, writing review & editing.

## Supporting information



Supporting Information

## Data Availability

The data that support the findings of this study are available from the corresponding author upon reasonable request.
