# Supplementary material for: High Specific Strength Eutectic High‐Entropy Alloy: Collaborative Effects of TRIP, TWIP, and Nanoprecipitation
Source: Adv Sci (Weinh). 2025 Apr 30;12(27):2501703. doi: 10.1002/advs.202501703 (PMC12279185; doi:10.1002/advs.202501703)
Supplement: Supplementary file 1 — Supporting Information [file ADVS-12-2501703-s001.docx]

**Supporting Information for**

**High specific strength eutectic high-entropy alloys: Collaborative effects of TRIP, TWIP and nanoprecipitation**

Z. Q. Wang ^a,b^, X. T. Li ^a^, Z. J. Zhang ^a,b^ and Z. F. Zhang ^a,b*^

*^a^ Shenyang National Laboratory for Materials Science, Institute of Metal Research, Chinese Academy of Sciences, Shenyang 110016, P.R. China.*

*^b^ School of Materials Science and Engineering, University of Science and Technology of China, Shenyang 110016, China*

* Corresponding author. Email: [zhfzhang@imr.ac.cn](mailto:zhfzhang@imr.ac.cn)

**Section S1** **The process of composition design**

For metallic materials, enhancing the work-hardening ability is crucial to improve overall mechanical properties. Comparing to the widely studied AlCoCrFeNi_2.1_ eutectic high-entropy alloy (EHEAs), We chose to exclude Fe element because it has moderate mixing entropy, mixing enthalpy, valence electron concentration (VEC), and stacking fault energy (SFE) [1, 2], which have little effect on the formation of heterogeneous phases and mechanical properties. Excluding Fe is conducive to have greater compositional adjustment space. CoCrNi system medium-entropy alloys (MEAs) have been extensively studied and proven to exhibit excellent mechanical properties with adjustable compositions in a wide range [3, 4]. Therefore, we selected to introduce eutectic phases into CoCrNi system MEAs for further improvement of mechanical properties. As is well known, the combination of Al and Ni elements possesses the strongest binding energy and the lowest mixing entropy (i.e. -22 kJ/mol) [2], making the addition of Al beneficial for the introduction of the NiAl B2 phase structure. Wu *et al.* [5] summarized the relationship between the formation of eutectic structures and VEC, noting that most EHEAs occur at a VEC of 7.5-8.0. Furthermore, the constituent elements of the alloy can be categorized into three types: 1) Al as the critical element for eutectic formation, 2) Cr as associated element, and 3) the remaining Fe, Co, Ni as miscible elements. A distribution map illustrating the relationship between the eutectic range and the content of Al and Cr elements was also provided. Based on the analysis above, we chose an Al content of 17% (the reason will be explained in subsequent sections) and set the Cr content to 20%. This decision was based on two considerations. Firstly, we selected an FCC: BCC volume fraction ratio of 6:4. On the one hand, Lu *et al.* [6] demonstrated that 6:4 is an ideal ratio in EHEAs. On the other hand, according to our experimental results, as shown in Figure S1a, it is indicated that when the volume fraction ratios were 7:3, 6:4, and 5:5, corresponding to Al contents of 19%, 17%, and 15.5%, respectively, Al17 exhibited the best mechanical properties. We infer that an FCC: BCC volume fraction ratio of 6:4 may yield the optimal heterogeneous deformation-induced (HDI) strengthening effect, and the different morphologies of B2 phase at different ratios may have certain effects (Figure S1b). Secondly, to enhance the mechanical properties of each phase, the SFE needs to be reduced to activate more work-hardening mechanisms. Although Ni has the largest mixing enthalpy with Al, it also has the highest SFE (i.e. 125 mJ/m^2^) [7] among the several matrix elements. Conversely, Co has a slightly lower mixing enthalpy with Al (i.e. -19 kJ/mol) [2] than Ni, can also form a heterogeneous structure, and possesses the lowest SFE (i.e. -1 mJ/m^2^) [8, 9]. Xu *et al.* [10] presented the two-phase composition of a cast CoCrNiAl EHEA, which we used as a basis for optimization. The Al and Cr contents were fine-tuned to ensure the formation of a eutectic structure, while the Co and Ni contents were adjusted to reduce the SFE. As shown in Figure S1c, a high Ni content does not achieve the desired strengthening effect due to inhibited work-hardening ability. Based on the above analysis, we determined the two-phase compositions as FCC: FCC phase: Co 38 %, Cr 27 %, Ni 26 %, Al 9 %; B2 phase: Co 24 %, Cr 10 %, Ni 36 %, Al 30 %. The overall composition can be derived from 0.6(Co38Cr27Ni26Al9) and 0.4(Co24Cr10Ni36Al30), finally we obtained the overall composition as Co_32.4_Cr_20.2_Ni_30.0_Al_17.4_. After rounding, the nominal composition became Co_33_Cr_20_Ni_30_Al_17_ (at%).

**Section S2 MATERIALS AND METHODS**

**Sample preparation**

The Co_33_Cr_20_Ni_30_Al_17_ (at%) EHEA was fabricated through vacuum induction melting in an Ar-gettered argon atmosphere (purity lager than 99.95%). The ingots were remelted at least for five times to ensure the chemical homogeneity. The as-cast alloy was homogenized at 1250 ℃ for 4 hours followed by water quenching and cold rolling at room temperature with a total reduction of 70 %, noting that the amount of pressure applied per pass should not exceed 0.05 mm. Finally, the rolled EHEA was annealed at 810 ℃, 730 ℃, 670 ℃ for 1 h, and the annealed samples are referred as MMAS810, MMAS730, MMAS670, respectively.

**Microstructural characterization**

The density of sample was measured by Archimedes' method using a density balance (XS105). The phase transformation temperatures were measured by a differential scanning calorimeter (DSC, STA449F3). A constant heating and cooling rate of 20 ℃/min was used for the thermal analysis. The sample surfaces were polished using a 2000# grit SiC paper and electro-chemically polished using a solution of HClO4: CH3COOH = 1: 9 (Vol.) under a voltage of 20 V for 20 s at -20 ℃ to obtain mirror finish. The Electron backscattered diffraction (EBSD) orientation mappings and electron channeling contrast images (ECCI) were conducted using a ZEISS Sigma-500 field emission scanning electron microscope (SEM) with an operating voltage of 20 kV. The high-resolution transmission electron microscopy (HR-TEM) with high-angle annular dark-field scanning transmission electron microscopy (HAADF-STEM) pattern and Energy Dispersive X-ray Spectroscopy (EDX) were conducted by Thermofisher Talos F200X. The TEM foils were prepared using a twin-jet electropolishing method by Tenupole-5 in a solution of 10 % perchloric and acid 90% alcohol, with a voltage of 20 V and temperature of -20 ℃.

**Tensile test**

The uniaxial tensile tests were carried out on an Instron 8862 instrument at room temperature with a constant strain rate of 1 × 10^-3^ s^-1^. The strain was measured using the digital image correlation (DIC) technique. The dog-bone-shaped tensile samples with a gauge section dimension of 6 × 3 × 1.2 mm^3^ were prepared using electrical discharge machining and 2000# SiC paper to polish for removing oxide scale from the samples. All tensile tests were repeated at least for three times to ensure the repeatability and credibility.

**The calculation of SUTS**

Specific ultimate tensile (Yield) strength is defined as the ratio of ultimate tensile (Yield) strength to density. The density (ρ) of MMAS EHEA and data in Table S2 were calculated by the regression formula [11]:

$$\frac{1}{\rho}=\sum_{i} \frac{1}{\rho_{i}}C_{i}^{wt}(x)$$

where $\rho_{i}$ was the density of element i, and the $C_{i}^{wt}\left( x \right)$ was the mass content of element i.


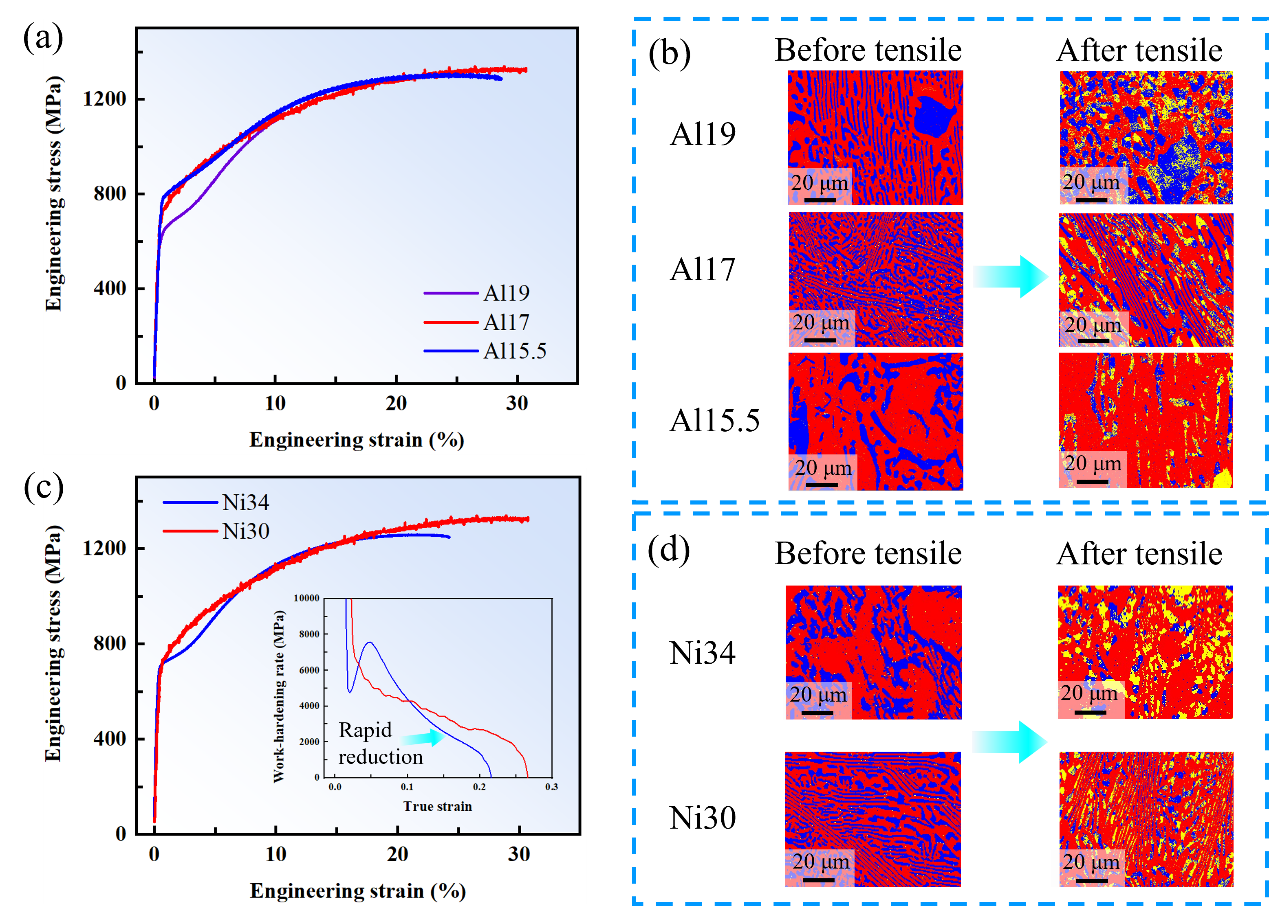


Figure S1. Mechanical properties and EBSD phase maps before and after tensile tests of different components annealed at 1100 ℃ for 5 mins. (a) Tensile test results and (b) EBSD phase maps before and after tensile with FCC: BCC ratios of 7:3, 6:4, 5:5; (c) Tensile test results and (d) EBSD phase maps before and after tensile with high and low Ni content.


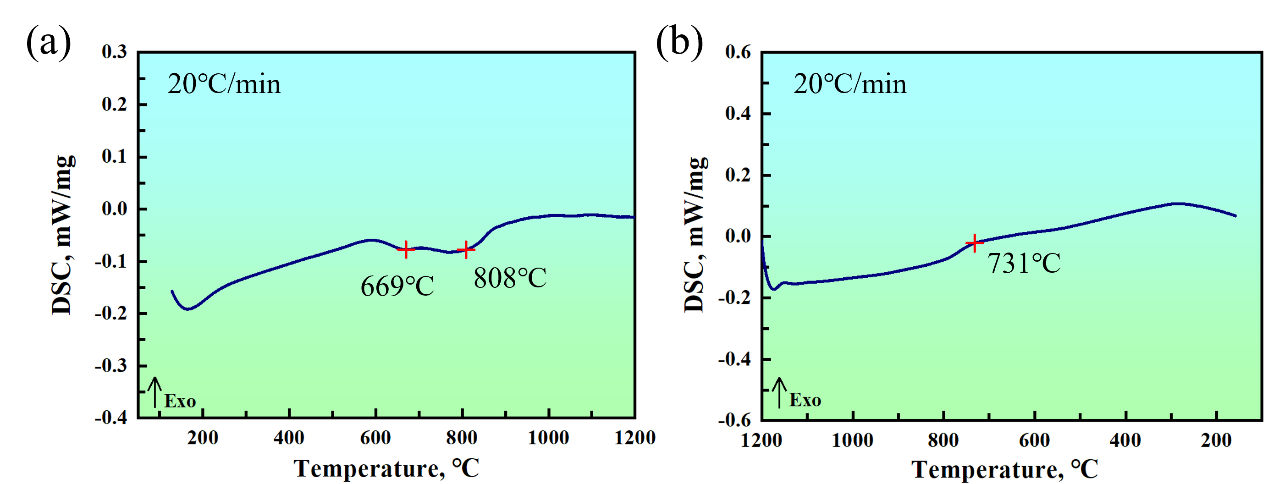


**Fig. S2. Characteristic temperature point indicated by the DSC curve for the as-cast EHEA** (a) cycle heating curve; (b) cycle cooling curve.


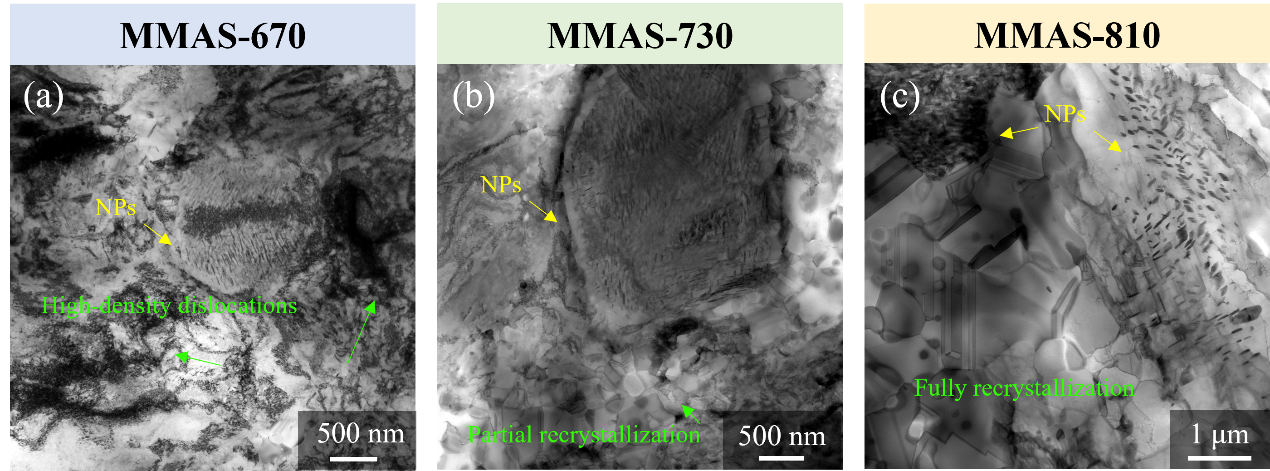


**Figure S3. The characteristics of the FCC matrix at different heat treatment temperatures.** (a) High-density dislocations at MMAS670. (b) Partial-recrystallization at MMAS730. (c) Fully-recrystallization at MMAS810.


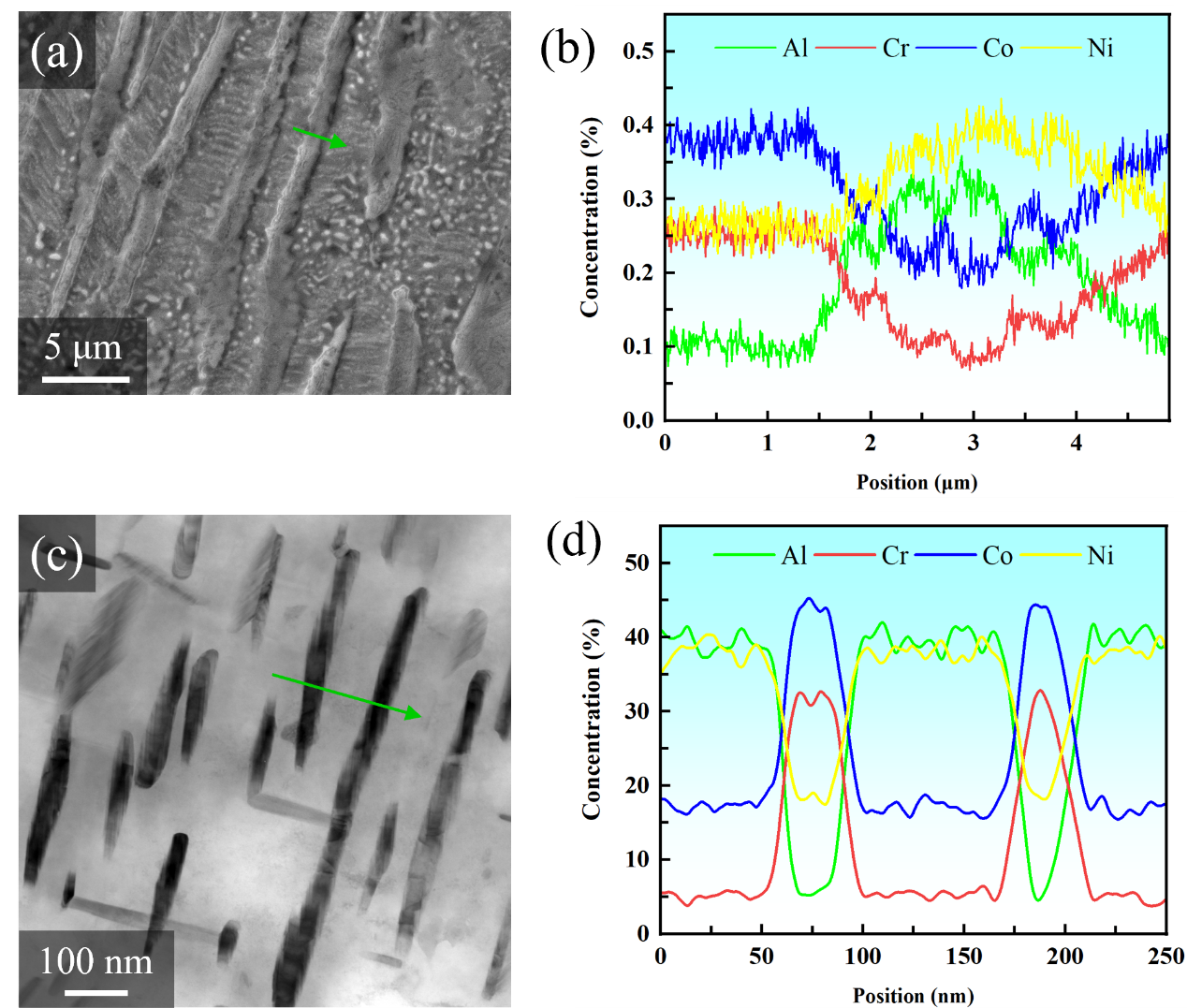


**Figure S4.** (a) SEM image showing dual-phase structure of EHEA. (b) The corresponding element profile across FCC/BCC phases; (c) TEM bight field (BF) image of blue frame in Fig. 1(d); (d) the corresponding EDX composition profiles.


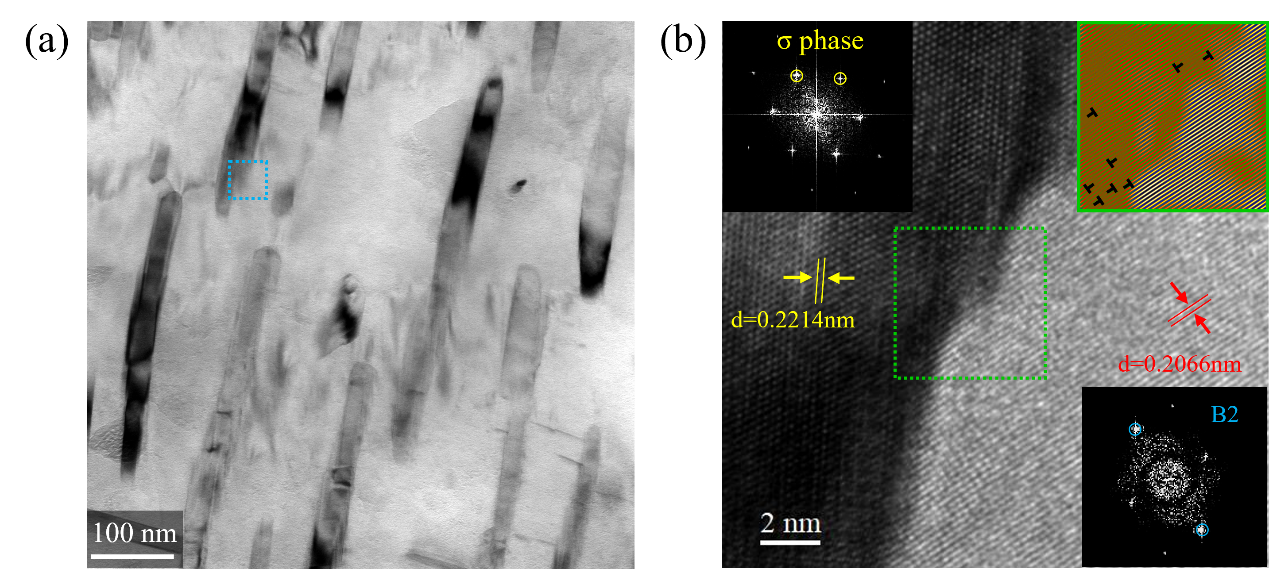


**Figure S5.** (a) The nanoprecipitations of MMAS810 samples. (b) The details of enlarged images marked by blue frame in (a), the corresponding fast Fourier transform (FFT) pattern proved that the left side is the σ phase and the right side is the NiAl B2 phase, the inverse Fourier transform (IFFT) pattern corresponding to the green frame can observe the geometrical necessary dislocations (GNDs) generated to maintain the continuity of the semi-coherent interface.


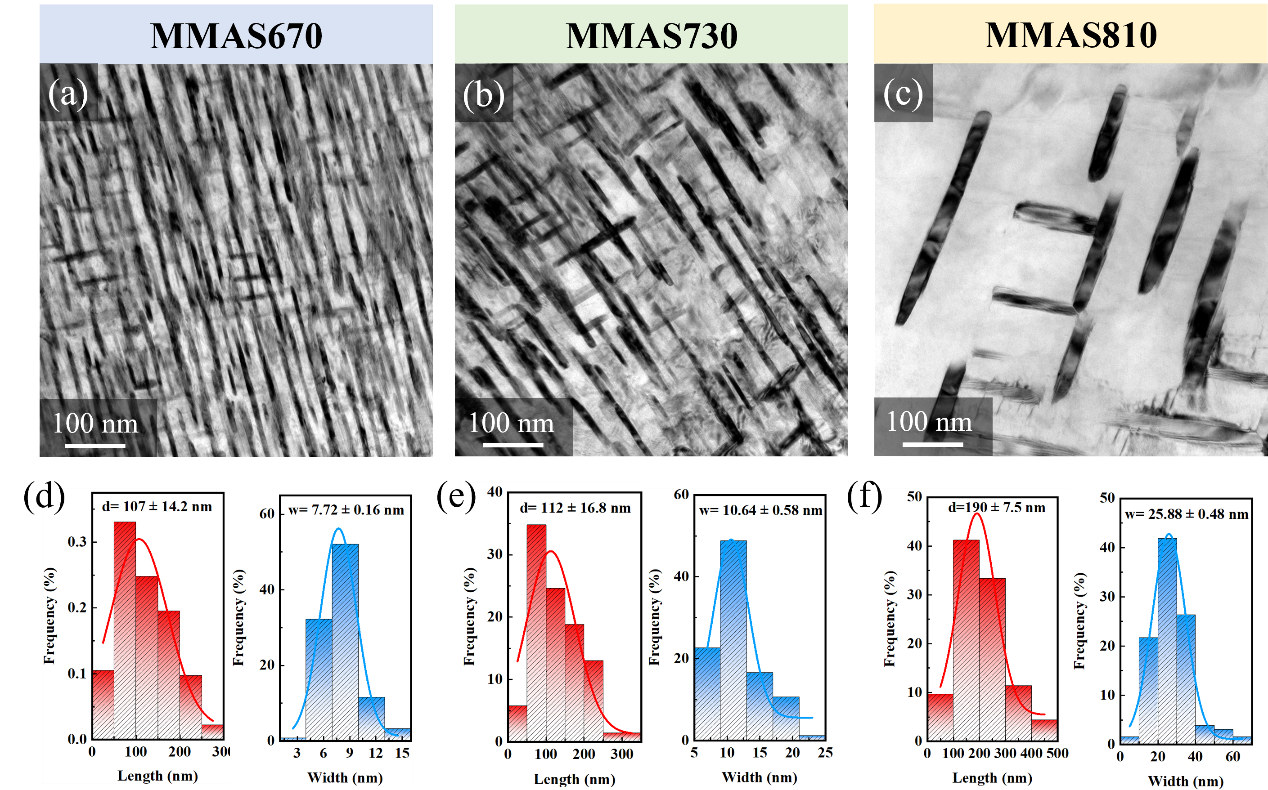


**Figure S6. The characteristics of the nanoprecipitations (NPs) under different heat treatment temperatures.** (a) MMAS670; (b) MMAS730; (c) MMAS810; (d-f) quantitative statistics of length and width corresponding to (a-c), respectively. (Supplementary explanation: in order to more accurately record the data of MMAS810, we collected more data from other images).


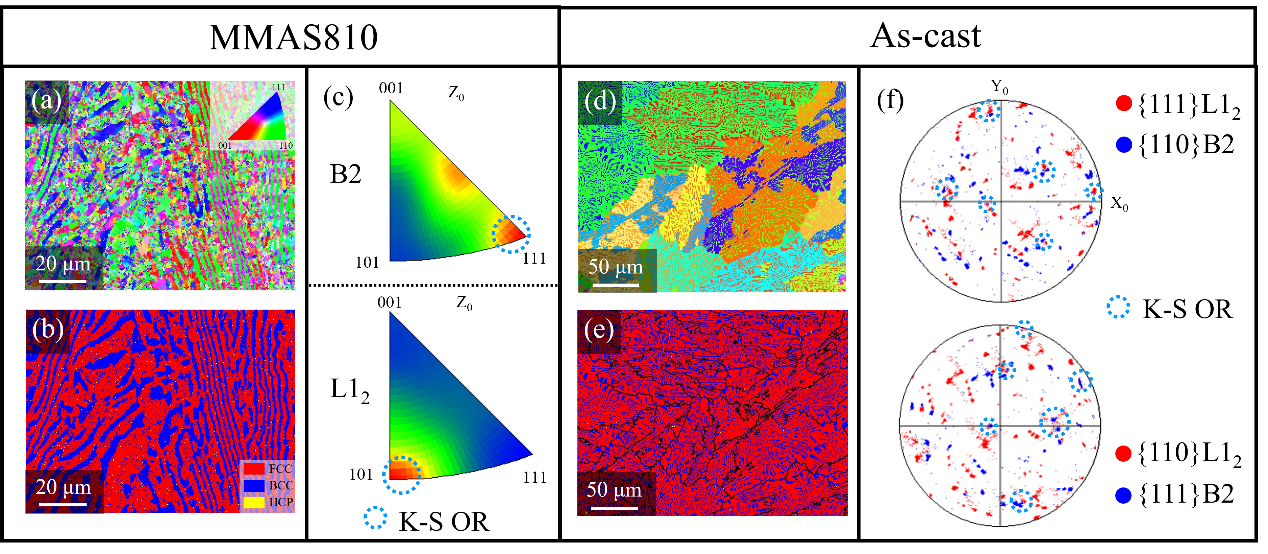


**Figure S7. Texture characteristics and orientation for as-cast and MMAS810 samples** (a) Inverse pole figure (IPF) and (b) phase map of MMAS810 sample. (c) IPF image showing the K-S orientation relationship (OR) between {111}_B2_ and {101}_L12_ in MMAS810 sample. (d) IPF figure (e) phase maps of as-cast sample. (f) Pole figure (PF) reveals the K-S OR of L1_2_ and B2 phase in as-cast sample.


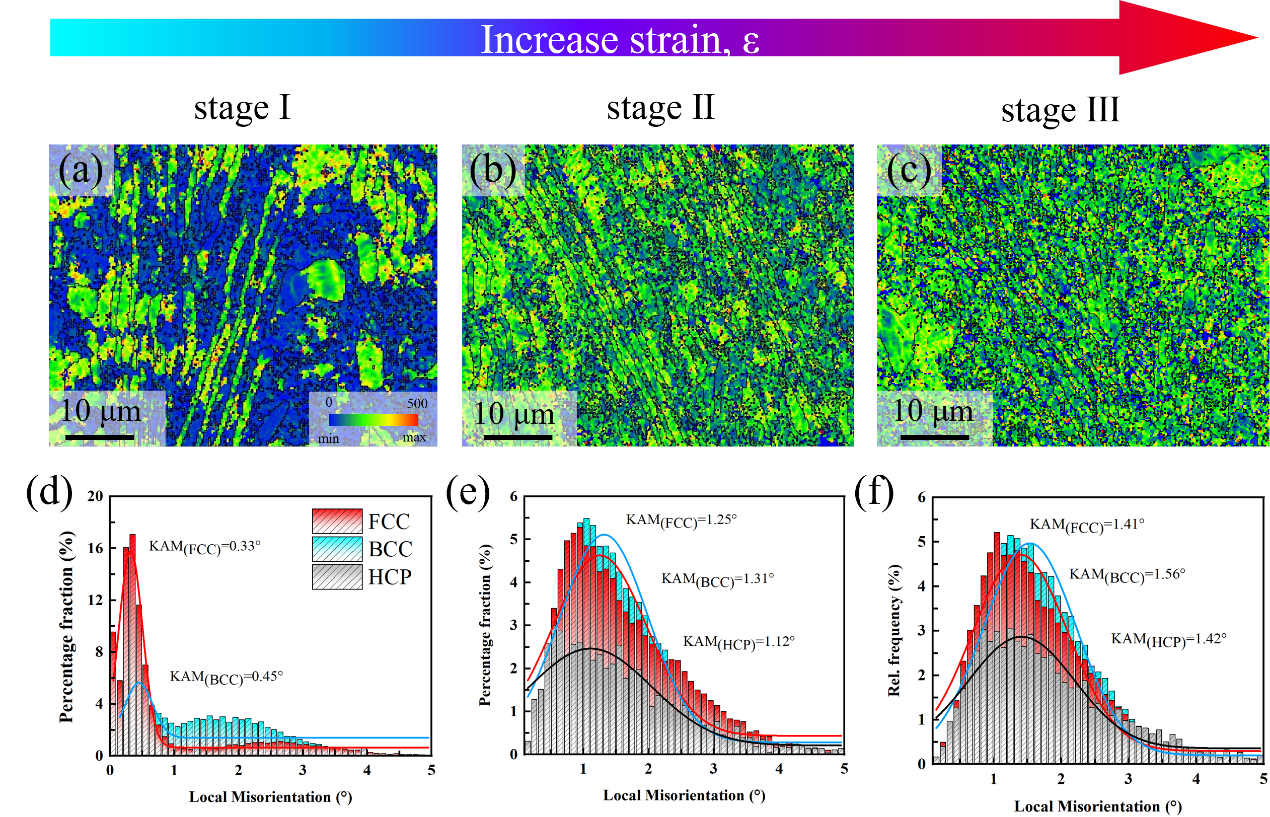


**Figure S8.** (a-c) The Kernel average misorientation (KAM) maps corresponding to Fig. 3(a_ⅰ_-a_ⅲ_), respectively. (d-f) Quantitative statistics of KAM data corresponding to (a-c), the local strain increases gradually with the increase in deformation, and the FCC phase is always slightly higher than the BCC phase.


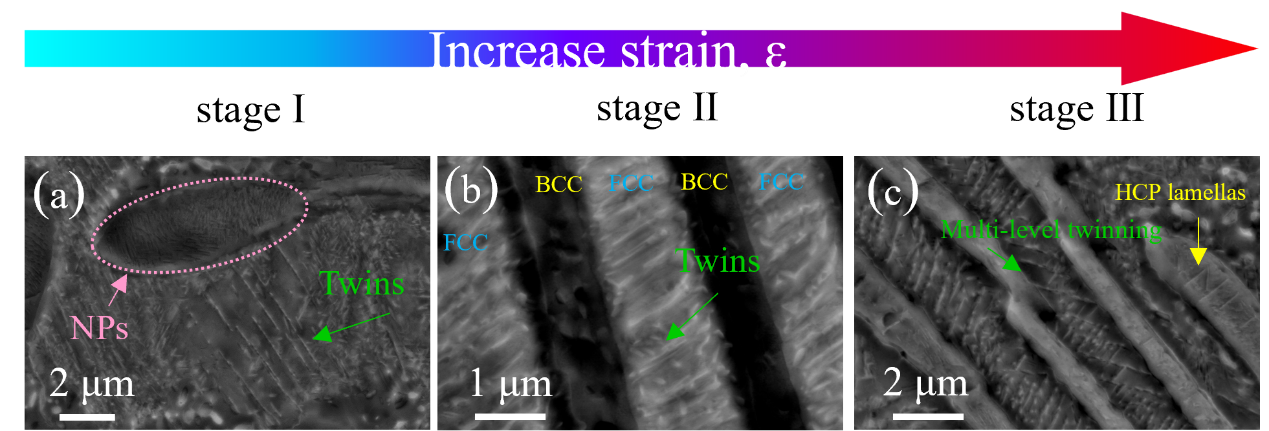


**Figure S9. Electron channeling contrast (ECC) images revealed the evolution of deformation twinning at three stages**. The evolution and transition process of twinning from a small number of twins (a) parallel twins (b) multi-level twins (c) high-density NPs and HCP lamellas observed in the BCC grains in (a) and (c), respectively.


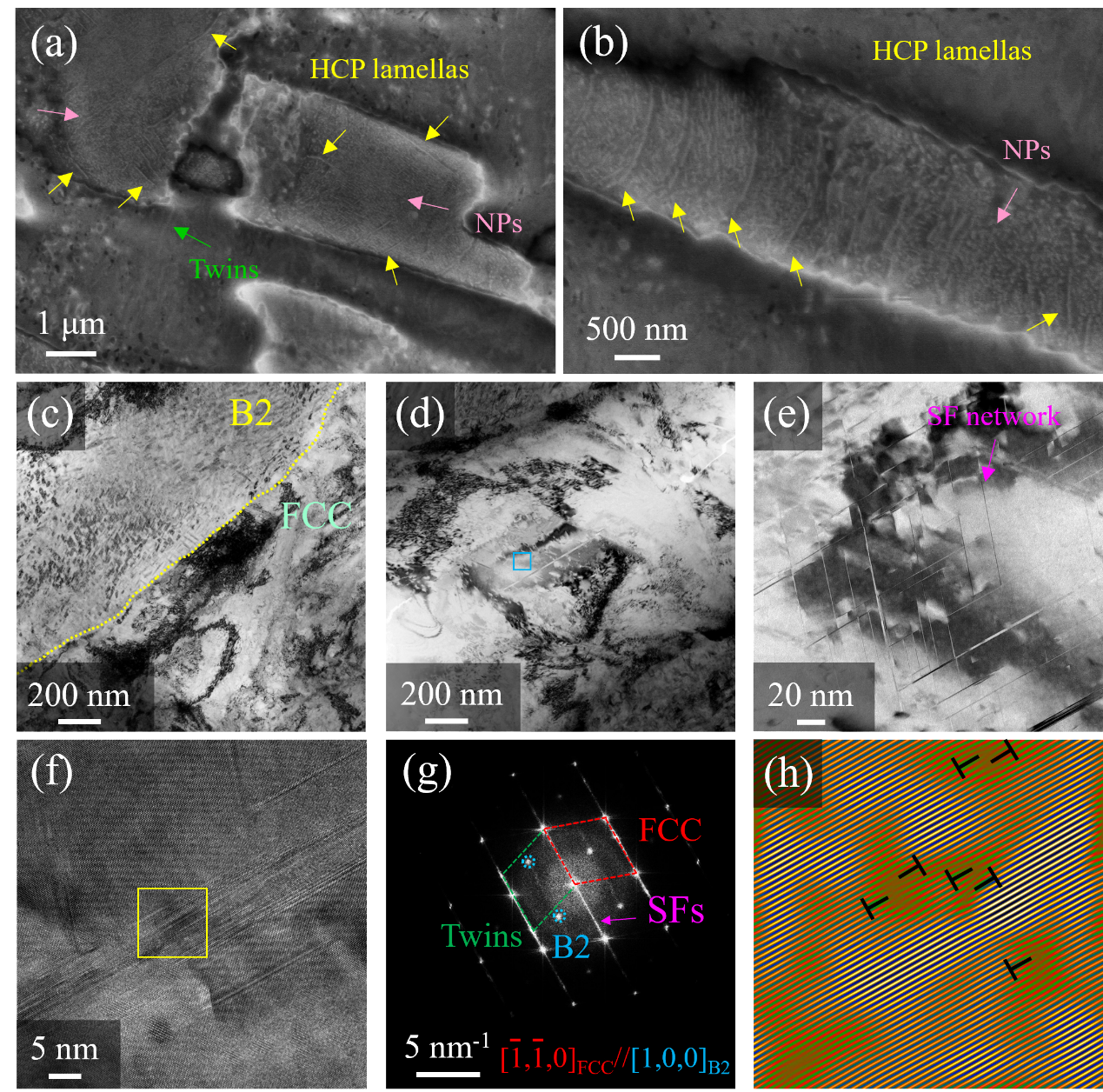


**Figure S10. Deformation feature and damage microstructures after tension of MMAS670 sample.** (a) HCP lamellas and nanoprecipitations in B2 phase. (b)Enlarged image showing high-density HCP lamellas and nanoprecipitations. High-density dislocations existing in (c) B2 phase and (d) FCC matrix. (e) Enlarged image of blue frame in (d) showing SF networks in FCC phase. The HR-TEM image (f) and corresponding to FFT pattern (g) and IFFT pattern (h) proved the stacking state of SFs, nanotwins, and dislocations. TWIP effect improved the ductility of A670 sample.


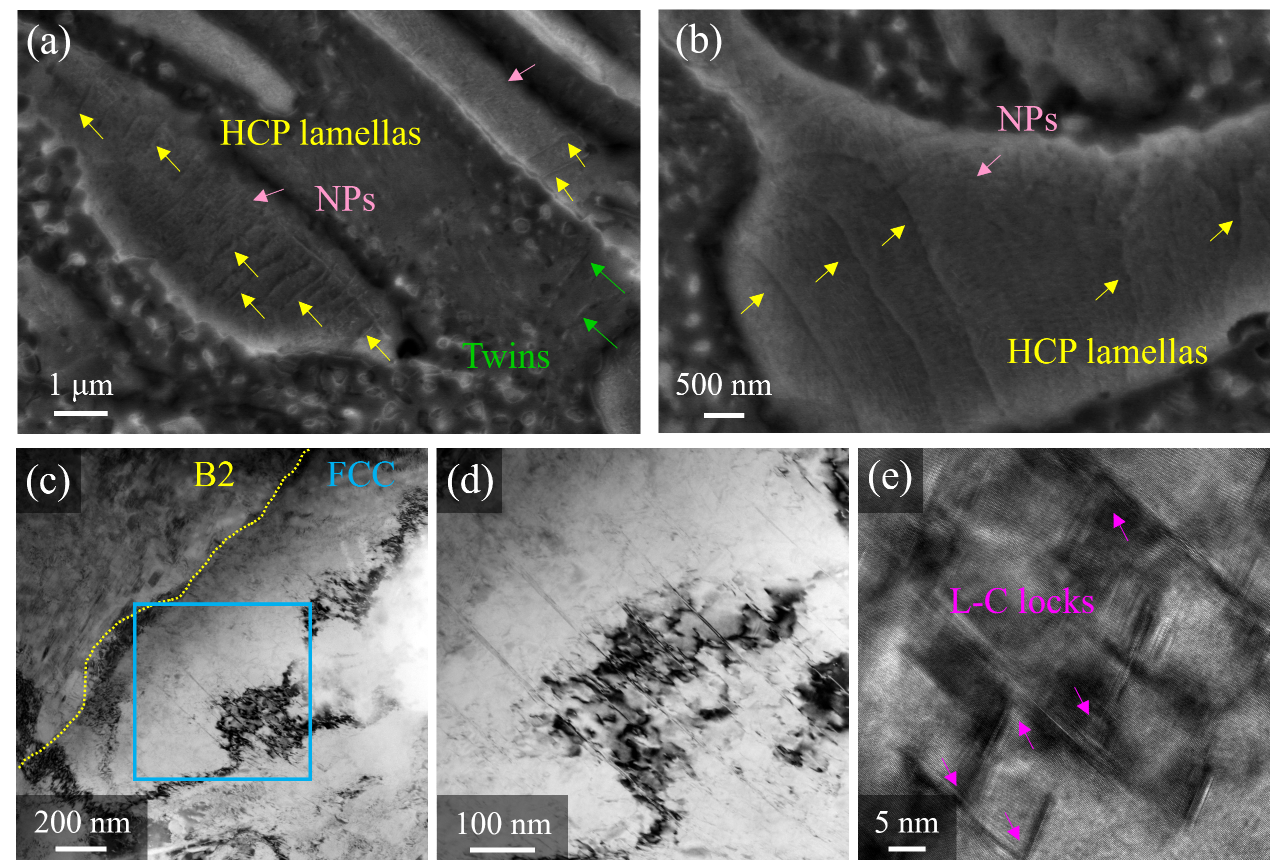


**Figure S11. Deformation feature and damage microstructures after tension of MMAS730 sample.** (a) Collaborative strength effects of TRIP, TWIP and nanoprecipitations. (b) HCP lamellas in B2 phase. (c) Piling-up of dislocations in both B2 and FCC phase. (d) Deformation twins in FCC matrix. (e) HR-TEM image showing SFs and L-C locks.


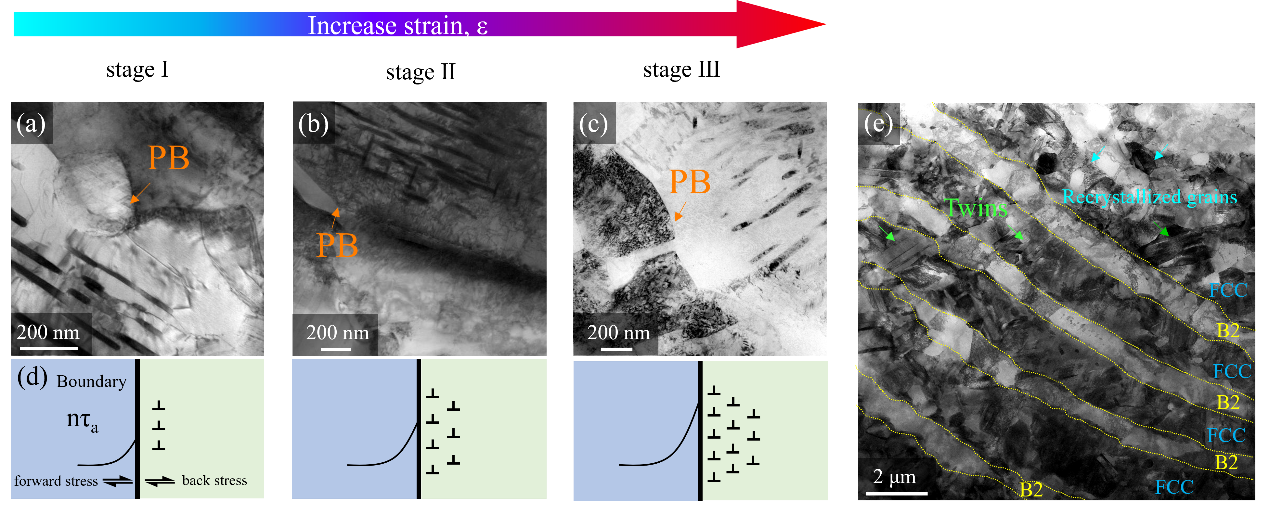


**Figure S12. The characteristics of dislocations in heterostructure.** (a-c) The hindering effect of phase boundary (PB) on dislocation motion at three stages. (d) Schematics of GNDs piled-up in phase boundary, illustrated the interaction between hard domain and soft domain [12]. (e) The mechanism characteristics of dual-phase structure after fracture, soft phase deformation along the gap of hard phase, recrystallization grains and twinning observed in the FCC matrix again.


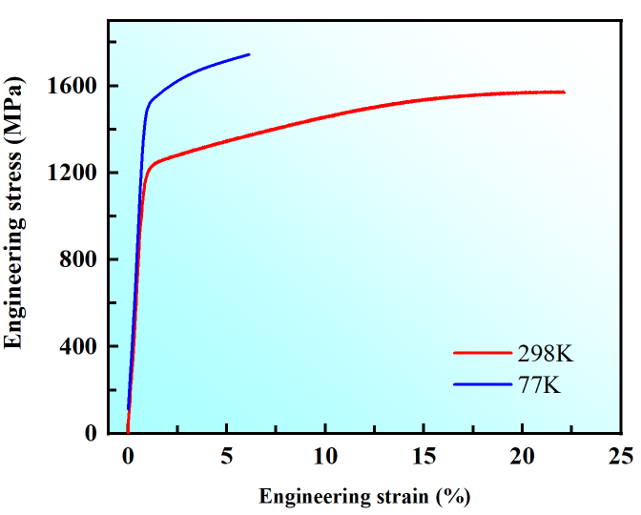


**Figure S13. Cryogenic tensile stress-strain curves of MMAS810 sample showing early catastrophic fracture at liquid nitrogen temperature.**


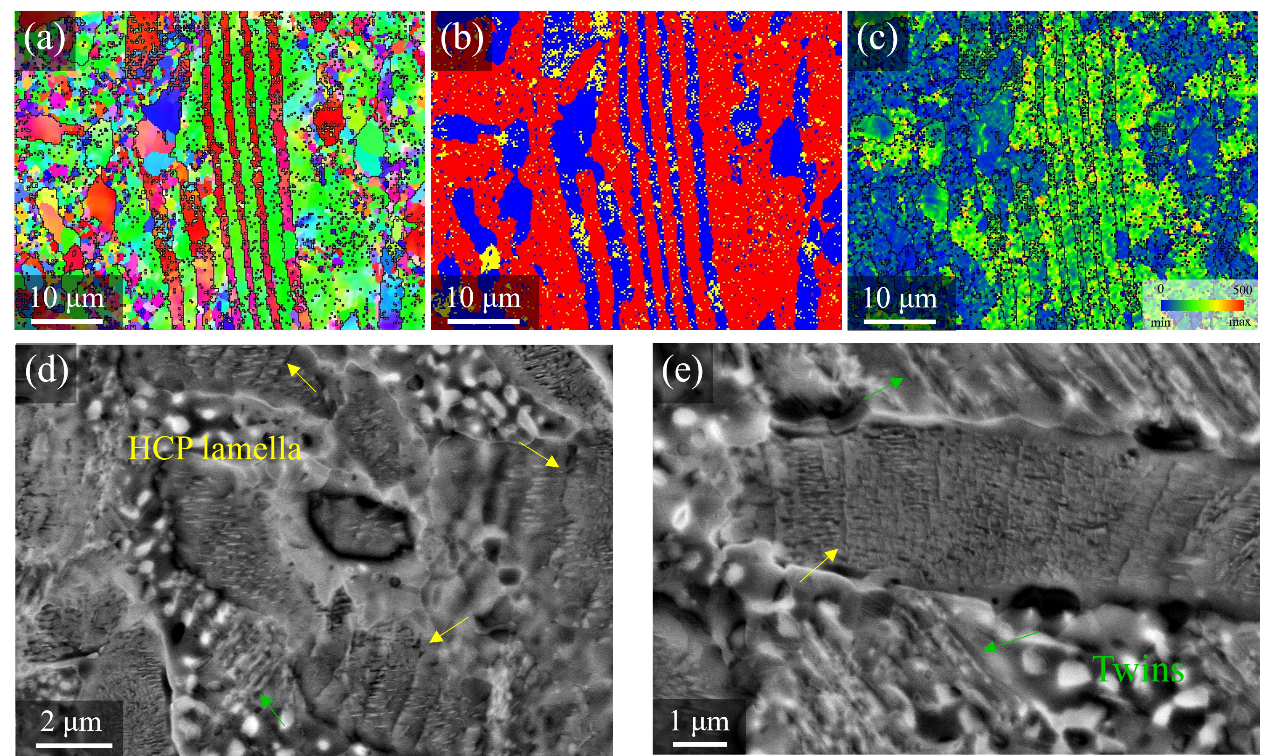


**Figure S14. Deformation feature and damage microstructures after tensile test of MMAS810 sample at 77K.** (a) IPF image and corresponding to (b) phase map and (c) KAM map show microstructure characteristics after tensile deformation. (d) and (e) ECC images showing the HCP lamellas in B2 phase and twins in FCC phase. no significant difference in deformation mechanism compared to ambient temperature.


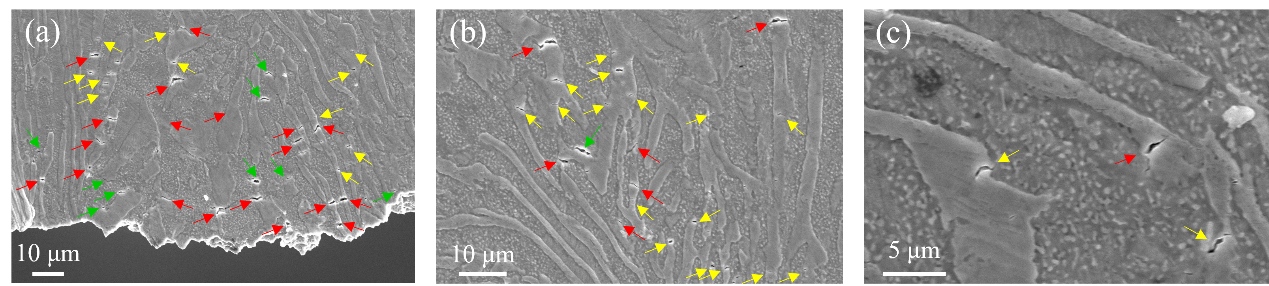


**Figure S15. SEM images of crack characteristics on the specimen surface of MMAS-810 sample.** (a) Near the fracture surface. (b) stay away from the fracture surface; (c) high-magnification HR-SEM image showing microscopic morphology of cracks; most of cracks penetrated the B2 phase (marked by red arrow), while the rest cracks along the phase boundary (marked by green arrow) or insert into the B2 phase (marked by yellow arrow), illustrated the fracture mode of B2 phase is crack initiation.


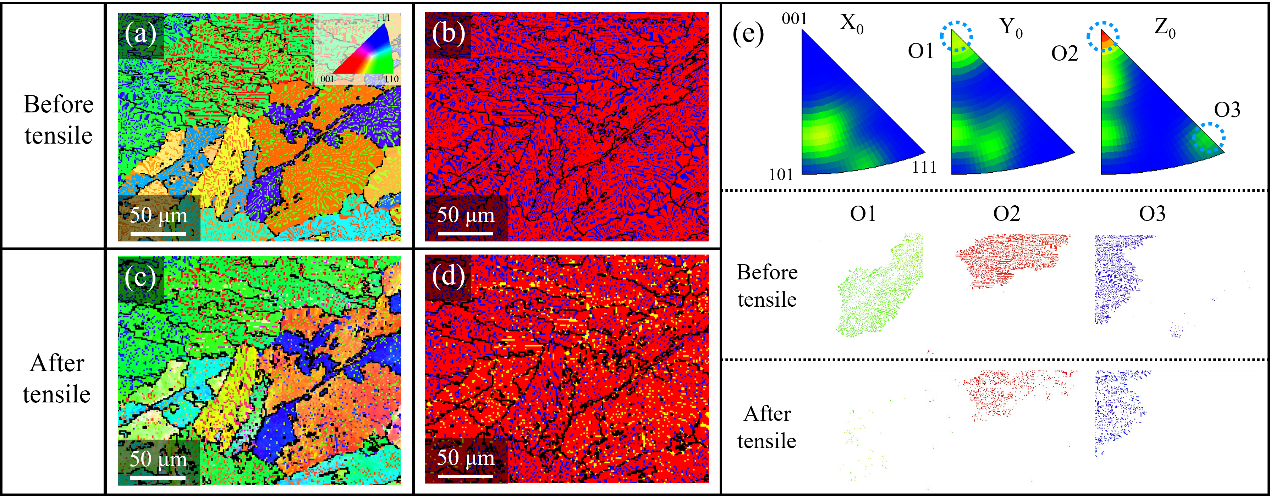


**Figure S16. Quasi in-situ tensile test showing microstructures evolution before and after tensile test of AC sample at ambient temperature.** (a) IPF image and corresponding to (b) phase map and (c) KAM map showing microstructure characteristics before tensile deformation. (d) IPF image and corresponding to (e) phase map and (f) KAM map showing microstructure characteristics after tensile deformation. The (110) orientation in the as-cast state is prone to phase transformation, leading to early catastrophic fracture.

**Table S1. Detailed data of Figs. 4a and b, including yield strength (YS), ultimate tensile strength (UTS), uniform elongation (UE) of this work and other eutectic high-entropy alloys in the literature available.**

| Composition | YS (MPa) | UTS (MPa) | UE (%) | Symbol | Reference |
| --- | --- | --- | --- | --- | --- |
| This work | 1773 | 2045 | 6.3 | 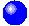 | **/** |
|  | 1587 | 1909 | 10.4 |  |  |
|  | 1218 | 1571 | 22.04 |  |  |
| AlCo0.4CrFeNi2.7 | 706 | 1139 | 21.0 | 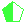 | [13] |
|  | 1039 | 1211 | 23.7 |  |  |
|  | 939 | 1289 | 17.3 |  |  |
|  | 1233 | 1372 | 14.2 |  |  |
|  | 1228 | 1444 | 14.2 |  |  |
|  | 1217 | 1500 | 16.1 |  |  |
| Ni30Co30Fe10Cr10Al18W2 | 808 | 1266 | 20.3 | 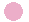 | [5] |
| Ni36Co24Fe10Cr10Al18W2 | 851 | 1316 | 19.7 |  |  |
| Ni40Co20Fe10Cr10Al18W2 | 890 | 1344 | 20.6 |  |  |
| Ni30Co30Fe10Cr10Al18W2 | 784 | 1368 | 35 | 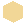 | [14] |
| Ni49Fe28Al17V6 | 760 | 1290 | 26.0 | 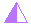 | [15] |
| AlCoCrFeNi2.1 | 1740 | 1802 | 4.1 | 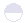 | [16] |
|  | 1113 | 1190 | 11.1 |  |  |
|  | 1437 | 1526 | 14.0 | 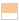 | [17] |
|  | 1100 | 1175 | 11.0 |  |  |
|  | 1490 | 1638 | 15.2 | 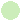 | [18] |
|  | 1263 | 1442 | 20.4 |  |  |
|  | 1154 | 1340 | 22.7 |  |  |
|  | 1003 | 1286 | 20.5 | 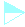 | [19] |
|  | 1333 | 1640 | 13.6 |  |  |
| AlCoCrFeNi2.1 | 1615 | 1907 | 7.5 |  |  |
|  | 1015 | 1270 | 22.5 | 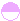 | [20] |
|  | 1028 | 1284 | 21.6 |  |  |
|  | 1102 | 1358 | 9.3 |  |  |
|  | 1304 | 1445 | 6.1 |  |  |
|  | 1344 | 1499 | 6.3 |  |  |
|  | 691 | 1009 | 14.4 | 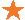 | [21] |
|  | 645 | 922 | 13.8 |  |  |
|  | 470 | 691 | 18.3 |  |  |
|  | 428 | 696 | 32.4 |  |  |
|  | 1108 | 1200 | 12 | 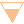 | [22] |
|  | 844 | 1175 | 23 |  |  |
|  | 648 | 1075 | 27 |  |  |
|  | 926 | 1361 | 10.2 | 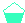 | [23] |
| Co30Cr10Fe10Al18Ni32 | 752 | 1129 | 16.7 | 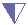 | [24] |
| Co30Cr10Fe10Al18Ni31Mo1 | 763 | 1160 | 18.2 |  |  |
| Co30Cr10Fe10Al18Ni30Mo2 | 688 | 1251 | 14.7 |  |  |
| Co30Cr10Fe10Al18Ni29Mo3 | 700 | 1198 | 14.5 |  |  |
| Fe20Co20Ni41Al19 | 577 | 1103 | 18.7 | 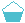 | [25] |
| Al0.7CoCrFeNi | 723 | 1158 | 35.4 | 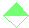 | [26] |
| Al0.9CoFeNi2 | 559 | 1005 | 6.2 | 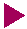 | [27] |
| AlCoCrNi2 | 553 | 1200 | 8.9 | 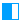 | [28] |
| Al0.9CoCrNi2.1 | 645 | 1033 | 6.9 |  |  |
|  | 820 | 1400 | 11.6 |  |  |
| Al0.8CoCrNi2.2 | 578 | 969 | 17.3 |  |  |
| Al0.7CoCrNi2.3 | 518 | 853 | 26.2 |  |  |
| AlCoCrFeNi2 | 546 | 1076 | 16.6 | 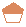 | [29] |
| AlCoCrFeNi2.1 | 546 | 1046 | 17.7 |  |  |
| AlCoCrFeNi2.2 | 544 | 1120 | 20.5 |  |  |

**Table S2. Detailed data of Fig. 4c, including specific yield strength (SYS), specific ultimate tensile strength (SUTS), uniform elongation (UE) of this work and other high-entropy alloys or lightweight engineering metallic alloys in the literature. (****Our density values are derived from the Archimedes' method measurements, while the density values of other components from literatures are calculated using theoretical formulas as shown** **in the calculation of SUTS section).**

| Composition | SYS (MPa/gcm^-3^) | SUTS (MPa/gcm^-3^) | UE (%) | Density  (gcm^-3^) | | Reference |  |
| --- | --- | --- | --- | --- | --- | --- | --- |
| This work | 240 | 273 | 6.3 | | 7.49 | **/** | |
|  | 214 | 258 | 10.4 | |  |  |  |
|  | 164 | 212 | 22.04 | |  |  |  |
| eutectic high-entropy alloy (AlCoCrFeNi2.1) | 189 | 233 | 13.6 | | 7.05 | [19] | |
|  | 142 | 182 | 20.5 | |  |  |  |
|  | 211 | 232 | 15.2 | | 7.05 | [18] | |
|  | 179 | 205 | 20.4 | |  |  |  |
|  | 157 | 169 | 11 | | 7.05 | [16] | |
|  | 157 | 170 | 12 | | 7.05 | [22] | |
| CoCrNi | 126 | 132 | 21 | | 8.27 | [30] | |
|  | 115 | 127 | 27 | |  |  |  |
|  | 101 | 122 | 35 | |  |  |  |
|  | 98 | 112 | 22.5 | | 8.27 | [31] | |
|  | 64 | 98 | 37 | |  |  |  |
|  | 103 | 131 | 32.5 | | 8.27 | [32] | |
|  | 126 | 142 | 26.3 | |  |  |  |
| CoCrFeMnNi | 100 | 111 | 27 | | 8.01 | [33] | |
|  | 77 | 109 | 39 | | 8.01 | [34] | |
|  | 83 | 113 | 38 | |  |  |  |
|  | 94 | 115 | 34 | |  |  |  |
|  | 111 | 123 | 25 | |  |  |  |
|  | 101 | 111 | 30.1 | | 8.01 | [35] | |
|  | 77 | 108 | 38 | |  |  |  |
|  | 73 | 102 | 31.7 | | 8.01 | [36] | |
|  | 86 | 103 | 21 | | 8.01 | [37] | |
|  | 67 | 93 | 33 | |  |  |  |
| TC4 | 203 | 218 | 5.67 | | 4.43 | [38] | |
|  | 215 | 226 | 5.13 | |  |  |  |
|  | 205 | 223 | 4.56 | |  |  |  |
|  | 209 | 225 | 5.14 | |  |  |  |
|  | 262 | 277 | 3.93 | | 4.43 | [39] | |
|  | 246 | 267 | 3.32 | |  |  |  |
|  | 205 | 221 | 8.03 | |  |  |  |
|  | 239 | 266 | 6.19 | | 4.43 | [40] | |
|  | 215 | 248 | 7.74 | |  |  |  |
|  | 217 | 254 | 8.01 | |  |  |  |
|  | 213 | 235 | 9.47 | |  |  |  |
|  | 208 | 234 | 7.7 | |  |  |  |
|  | 216 | 237 | 5.94 | |  |  |  |
| 7075 Al alloy | 179 | 204 | 9 | | 2.80 | [41] | |
|  | 206 | 221 | 6 | |  |  |  |
|  | 217 | 229 | 8.2 | |  |  |  |
|  | 188 | 216 | 10.01 | | 2.80 | [42] | |
|  | 179 | 209 | 11.32 | |  |  |  |
|  | 152.87 | 198.98 | 14.02 | |  |  |  |
|  | 188 | 207 | 8.82 | |  |  |  |
|  | 164 | 197 | 13.21 | |  |  |  |
|  | 148 | 191 | 13.21 | |  |  |  |
|  | 182 | 198 | 9.02 | | 2.80 | [43] | |
|  | 240 | 251 | 7.82 | |  |  |  |
|  | 230 | 244 | 7.78 | |  |  |  |
|  | 240 | 253 | 8.4 | |  |  |  |
|  | 205 | 222.5 | 8.31 | |  |  |  |
| Mg alloy | 26 | 76 | 19 | | ~1.80 | [44] | |
|  | 78 | 139 | 28 | |  |  |  |
|  | 61 | 139 | 17 | |  |  |  |
|  | 33 | 111 | 30 | |  |  |  |
|  | 50 | 94 | 20 | |  |  |  |
|  | 65 | 118 | 30 | |  |  |  |
|  | 50 | 94 | 17 | |  |  |  |
|  | 50 | 88 | 18 | |  |  |  |
|  | 28 | 80 | 22 | |  |  |  |
|  | 83 | 127 | 19 | |  |  |  |
|  | 89 | 151 | 20 | |  |  |  |
|  | 68 | 142 | 24 | |  |  |  |
|  | 81 | 159 | 26 | |  |  |  |
|  | 106 | 169 | 25 | |  |  |  |
|  | 76 | 148 | 25 | |  |  |  |
|  | 81 | 137 | 28 | |  |  |  |
|  | 89 | 100 | 30 | |  |  |  |
|  | 91 | 142 | 19 | |  |  |  |
|  | 58 | 130 | 18 | |  |  |  |
|  | 67 | 122 | 23 | |  |  |  |
| FeMnAlC | 80 | 117 | 29.4 | | 6.89 | [45] | |
|  | 94 | 130 | 38.1 | | 6.77 |  |  |
|  | 125 | 141 | 35.7 | | 6.72 |  |  |
|  | 142 | 155 | 37 | | 6.58 | [46] | |
|  | 171 | 182 | 22 | | 6.56 |  |  |
|  | 134 | 154 | 28.5 | | 6.69 |  |  |
|  | 130 | 148 | 45 | | 6.52 |  |  |
|  | 159 | 161 | 24.6 | | 6.15 | [47] | |
|  | 149 | 153 | 38.4 | |  |  |  |
|  | 61 | 121 | 37.6 | | 6.79 | [48] | |
|  | 65 | 119 | 29.9 | |  |  |  |
|  | 175 | 165 | 33 | | 6.22 | [49] | |
|  | 159 | 138 | 38.1 | |  |  |  |
|  | 124 | 160 | 38.7 | | 6.58 | [50] | |

**References**

1. S.F. Liu, Y. Wu, H.T. Wang, J.Y. He, J.B. Liu, C.X. Chen, X.J. Liu, H. Wang, Z.P. Lu, *Intermetallics*, **2018**, 93, 269.
2. A. Takeuchi, A. Inoue, *Mater. Trans.* **2005**, 46, 2817.
3. D. Huang, Y. X. Zhuang, C. H. Wang, *Scr. Mater.* **2022**, 207, 114269.
4. H. W. Deng, M. M. Wang, Z. M. Xie, T. Zhang, X. P. Wang, Q. F. Fang, Y. Xiong, *Mater. Sci. Eng. A*, **2021**, 804, 140516.
5. Q. F. Wu, Z. J. Wang, X. B. Hu, T. Zheng, Z. S. Yang, F. He, J. J. Li, J. C. Wang, *Acta Mater.* **2020**, 182, 278.
6. W. J. Lu, X. Luo, Y. Q. Yang, J. T. Zhang, B. Huang, *Mater. Chem. Phys.* **2019**, 238, 121841.
7. C. B. Carter, S. M. Holmes, *Philos. Mag.* **2006**, 35, 1161.
8. B. S. Lee, Y. Koizumi, H. Matsumoto, A. Chiba, *Mater. Sci. Eng. A*, **2014**, 611, 263.
9. J. X. Yan , Z. J. Zhang , P. Zhang, J. H. Liu, H. Yu, Q. M. Hu, J. B. Yang, Z. F. Zhang, *J. Mater. Sci. Technol.* **2023**, 139, 232.
10. N. Xu, Y. B. Huang, X. M. Liu, D. P. Xuan, H. L. Lu, S. L. Li, Y. D. Wang, J. S. Wang, *Mater. Sci. Eng. A*, **2024**, 918, 147436.
11. U. Bohnenkamp, R. Sandström, *Steel Res.* **2000**, 71, 88.
12. Y. T. Zhu, X. L. Wu, *Mater. Res. Lett.* **2019**, 7, 393.
13. X. F. Song, Z. Z. Liu, P. K. Liaw, Y. Zhang, *Addit. Manuf.* **2024**, 85, 104171.
14. Q. F. Wu,F. He, J. J. Li, H. S. Kim, Z. J. Wang, J. C. Wang, *Nat. Commun.* **2022**, 13, 4697.
15. S. Feng, S. Guan, X.K. Liu, S.Y. Peng, K.W. Dong, Y. Yang, X.S. Chen, Y.Z. Liang, Q.P. Wang, Y.F. Liu, Y. Peng, K.H. Wang, W. Chen, J. Kong, *Mater. Res. Lett.* **2023**, 12, 26.
16. I. S. Wani, T. Bhattacharjee, S. Sheikh, Y. P. Lu, S. Chatterjee, P. P. Bhattacharjee, S. Guo N. Tsuji, *Mater. Res. Lett.* **2016**, 4, 174.
17. T. Bhattacharjee, I. S. Wani, S. Sheikh, I. T. Clark, T. Okawa, S. Guo, P. P. Bhattacharjee N. Tsuji, *Sci. Rep.* **2018**, 8, 3276.
18. P. J. Shi, W. L. Ren, T. X. Zheng, Z. M. Ren, X. L. Hou, J. C. Peng, P. F. Hu, Y.F. Gao , Y. B. Zhong, P. K. Liaw, *Nat. Commun.* **2019**, 10, 489.
19. J. Ren, Y. Zhang, D. X. Zhao, Y. Chen, S. Guan, Y. F. Liu, L. Liu, S. Y. Peng, F. Y. Kong, J. D. Poplawsky, G. H. Gao, T. Voisin, K An, Y. M. Wang, K. Y. Xie, T. Zhu, W. Chen, *Nature* **2022**, 608, 62.
20. Y. N. Guo a , H. J. Su a, H. T. Zhou, Z. L. Shen, Y. Liu, J. Zhang, L. Liu,H. Z. Fu, *J. Mater. Sci. Technol.* **2022**, 111, 298.
21. H. T. Zheng, R. R. Chen, G. Qin, X. Z. Li, Y. Q. Su, H. S. Ding, J. J. Guo, H. Z. Fu, *Intermetallics* **2019**, 113, 106569.
22. I.S. Wani, T. Bhattacharjee, S. Sheikh, P. P. Bhattacharjee, S. Guo, N. Tsuji, *Mater. Sci. Eng. A* **2016**, 675, 99.
23. T. Wang, M. Komarasamy, S. Shukla, R. S. Mishra, *J. Alloys Compd.* **2018**, 766, 312.
24. Z. S. Yang, Z. J. Wang, Q. F. Wu, T. Zheng, P. Zhao, J. K. Zhao, J. Y. Chen, *Appl. Phys. A* **2019**, 125, 208.
25. X. Jin, Y. Zhou, L. Zhang, X. Y. Du, B. S. Li, *Mater. Lett.* **2018**, 216, 144.
26. Y. Tang, H. K. Wang, X. P. Ouyang, C. Wang, Q. S. Huang, Q. K. Zhao, X. C. Liu, Q. Zhu, Z. Q. Hou, J. K. Wu, Z. C. Zhang, H. Li, Y.K. Yang, W. Yang, H. J. Gao, H. F. Zhou, *Nat. Commun.* **2024**, 15, 3932.
27. H. Jiang, D. X. Qiao, W. N. Jiao, K. M. Hana, Y. M. Lub, P. K. Liaw, *J. Mater. Sci. Technol.* **2021**, 61, 119.
28. X. L. Jin, Y. X. Bi, J. Li, B. S. Li, *Materialia* **2020**, 10, 100639.
29. Y. P. Lu, X. Z. Gao, L. Jiang, Z. N. Chen, T. M. Wang, J. C. Jie, H. J. Kang, Y. B. Zhang, S. Guo, H. H. Ruan, Y. H. Zhao, Z. Q. Cao, T. J. Li, *Acta Mater.* **2017**, 124, 143.
30. P. Sathiyamoorthi, J. W. Bae, P. A. Rad, J. M. Park,J. G. Kim, H. S. Kim, *Entropy* **2018**, 20, 849.
31. S. Yoshida, T. Bhattacharjee, Y. Bai, N. Tsuji, *Scr. Mater.* **2017**, 134, 33.
32. W. J. Lu, X. Luo, Y. Q. Yang, B. Huang, *Mater. Chem. Phys.* **2020**, 251, 123073.
33. Y. Z. Tian, S. J. Sun, H. R. Lin, Z. F. Zhang, *J. Mater. Sci. Technol.* **2019**, 35, 334.
34. S. J. Sun, Y. Z. Tian, H. R. Lin, X. G. Dong, Y. H. Wang, Z. J. Zhang, Z. F. Zhang, *Mater. Des.* **2017**, 133, 122.
35. S. J. Sun, Y. Z. Tian, H. R. Lin, X. G. Dong, Y. H. Wang, Z. J. Wang, Z. F. Zhang, *J. Alloys Compd.* **2019**, 806, 992.
36. M. V. Klimova, D. G. Shaysultanov, S. V. Zherebtsov, N. D. Stepanov, *Mater. Sci. Eng. A* **2019**, 748, 228.
37. G. H. Gu, R. E. Kim, J. Lee, H. S. Kim, *Mater. Sci. Eng. A* **2022**, 852, 143720.
38. B. H. Wang, L. Cheng, D. C. Li, *Materials* **2021**,14, 5329.
39. Z. Qu, Z. J. Zhang, R. Liu, L. Xu, Y. N. Zhang, X. T. Li, Z. K. Zhao, Q. Q. Duan, S. G. Wang, S. J. Li, Y. J. Ma, X. H. Shao, R. Yang, J. Eckert, R. O. Ritchie, Z. F. Zhang, *Nature* **2024**, 626, 999.
40. M. Simonelli, Y. Y. Tse, C. Tuck, *Mater. Sci. Eng. A* **2014**, 616, 1.
41. S. K. Panigrahi, R. Jayaganthan, *J. Alloys Compd.* **2011**, 509, 9609-9616.
42. S. V. Sajadifar, P. Krooß, H. Fröck, B. Milkereit, O. Kessler, T. Niendorf, *Metals* **2021**, 11, 1142.
43. M. H. Shaeri, M. T. Salehi, S. H. Seyyedein, M. R. Abutalebi, J. K. Park, *Mater. Des.* **2014**, 57, 250.
44. Z. Z. Jin, M. Zha, S. Q. Wang, S. C. Wang, C. Wang, H. L. Jia, H. Y. Wang, *J. Magnes. Alloy* **2022**, 10, 1191.
45. Y. Sutou, N. Kamiya, R. Umino, I. Ohnuma, K. Ishida,. *ISIJ Int.* **2010**, 50, 893.
46. M. G. Khouzani, J. R. McDermid, *Mater. Sci. Eng. A* **2015**, 621, 118.
47. G. Frommeyer, U. Brüx, *steel res. int.* **2016**, 77, 627.
48. A.Mondal, D.Pilone, A.Brotzu, F.Felli, *Procedia Struct. Integr.* **2021**, 33, 237.
49. S. Jeonga, G. Parka, B. Kima, J. Moonb, S. J. Parkb, C. Lee, *Mater. Sci. Eng. A* **2019**, 742, 61.
50. U. S. Ko, C. Kim, S. J. Park, H. U. Hong, *Int. J. Fatigue* **2024**, 181, 108149.
